# Pre-existing antibodies to candidate gene therapy vectors (adeno-associated vector serotypes) in domestic cats

**DOI:** 10.1371/journal.pone.0212811

**Published:** 2019-03-21

**Authors:** Pengfei Li, Eva Boenzli, Regina Hofmann-Lehmann, A. Katrin Helfer-Hungerbuehler

**Affiliations:** Clinical Laboratory and Center for Clinical Studies, Department of Clinical Diagnostics and Services, Vetsuisse Faculty, University of Zurich, Zurich, Switzerland; National Institute of Dental and Craniofacial Research, UNITED STATES

## Abstract

Adeno-associated virus (AAV) vectors represent promising candidates for gene therapy; however, pre-existing neutralizing antibodies (NAb) may reduce AAV vector delivery efficiency. In this study, the presence of AAV NAb was investigated in cats, which serve as a larger and outbred animal model for the prediction of gene therapy outcomes in humans but also in cats.Serum/plasma samples from 230 client-owned Swiss cats and 20 specified pathogen-free cats were investigated for NAb to AAV1, AAV2, AAV5, AAV6, AAV7, AAV8 and AAV9 using *in vitro* transduction inhibition and a beta-galactosidase assay. NAb to all tested AAV serotypes were found. Of the client-owned cats, 53% had NAb to one or more of the AAV serotypes. NAb (≥1:10) were found at frequencies of 5% (AAV6) to 28% (AAV7). The highest titers were found against AAV7 (≥1:160). The NAb prevalence to AAV2, AAV7 and AAV9 differed geographically. Regarding titers ≥1:10 against single AAV serotypes, age, breed and sex of the cats were not associated with the NAb prevalence. Cats with titers ≥1:20 against AAV2 and titers ≥1:40 against AAV7 were significantly younger than cats with low/no titers, and purebred cats were significantly more likely than non-purebred cats to have NAb to AAV2 (≥1:40). Additionally, regarding NAb to all AAV combined, female cats were significantly more likely than male cats to have NAb titers ≥1:40. Preliminary data using AAV-DJ indicated that less pre-existing NAb to the hybrid AAV-DJ can be expected compared to the wild-type AAV serotypes. AAV NAb will need to be taken into account for future *in vivo* gene therapy studies in cats.

## Introduction

Treatment of many genetic and acquired diseases remains a challenge. At present, curative treatments are often nonexistent, inefficient or toxic [1, 2]. Thus, new classes of therapeutics are being developed; these development efforts include the investigation and application of gene therapy. Adeno-associated virus (AAV)-based vectors represent promising candidates for therapeutic gene transfer due to the assumed lack of involvement in human diseases and their display of relatively low immunogenicity [3]. AAV has been used as a vector for over 20 years [4] in more than 100 clinical trials. Several of these trials show great promise, such as gene therapy for hemophilia B [5, 6], Leber’s congenital amaurosis [7, 8] and Parkinson’s disease [9]. Few AAV-vector-mediated gene therapies have already been approved in Europe including Alipogene Tiparvovec, a treatment of lipoprotein lipase deficiency as well as Luxturna a one-time gene therapy for the treatment of an inherited retinal dystrophy [10, 11].

With clinical assessment, however, some of the limitations of *in vivo* gene therapy have emerged. Both the safety and the efficacy of gene transfer *in vivo* using AAV vectors are affected by the host immune system [12]. Capsid-specific T-cell responses directed against transduced cells might limit the duration of transgene expression following AAV gene transfer [12]. In addition, previous infections with natural AAVs, which may have similar or even identical capsids, can result in the production of cross-reactive or specific anti-AAV neutralizing antibodies (NAb), which partially or even completely block transduction of the target tissue [5, 13, 14]. Thus, NAb directed against AAVs have a profound impact on transduction efficiency when the vector is delivered directly into the bloodstream or in any body compartment where immunoglobulin can be found [12]. In the last 50 years, numerous studies have investigated the seroprevalence of NAb directed against AAVs in the human population [15]. In humans, the presence of pre-existing neutralizing factors for AAV is generally high and varies geographically. By the age of two years, humans have already developed antibodies against different AAV virus capsids [16]. NAb prevalence can reach >70% for the serotype AAV2, which is the most prevalent serotype [15, 17, 18]. In comparison, NAb against AAV5, AAV6, AAV8, and AAV9 are significantly less prevalent, ranging from 15–47% of the population. However, methods to investigate NAb are difficult to standardize; thus, variations in prevalence among studies are inevitable [15, 18].

Animal studies and clinical trials have shown that low existing titers of NAb can hamper successful transduction and therefore negatively influence the outcome of gene therapy [13, 14, 19]. In addition, due to high sequence conservation, anti-AAV antibodies show a high degree of cross-reactivity across a wide range of serotypes. Strategies to overcome this humoral immunity to AAV are versatile, including selection of naïve subjects, using AAV serotypes with low seroprevalence, novel nonhuman primate AAV vectors and engineered chimeric AAVs, higher vector doses, transient immunosuppression, empty “decoy” capsids, and compartmentalized delivery [12, 14, 15, 20].

For the prediction of gene therapy outcomes in humans, accurate animal models are needed. Mouse models are commonly used and yield impressive results with efficient long-term transgene expression and low toxicity [21, 22]. However, when gene therapy is translated to larger animal models, such as cynomolgus macaques, the results are not as encouraging: transduction efficiencies are reduced and toxicity due to T-cell responses observed [23]. In addition, studies with non-human primates in general are controversial. Thus, other preclinical animal models are needed, preferentially without the complexity of nonhuman primate models. A potential candidate might be a cat model. In contrast to the laboratory mouse, the cat is an outbred animal with a long lifespan. Cats have already been used as animal models for several human diseases, such as Alzheimer’s disease [24], hereditary retinal blindness [25], cancer [26] and AIDS [27].

The prevalence of certain monogenic disorders within dog and cat breeds is substantial, probably driven by inbreeding and selection of desired characteristics. Gene therapy has already been successfully applied in cats for lysosomal storage disorders [28] such as the Sandhoff disease [29] or Mucopolysaccharidosis Type VI (MPS VI) [30]. Although treatment using gene therapy has not been used widely to cats, its potential for applications, such as the treatment of monogenic diseases in domestic cats, is tremendous.

Thus, by surveying pre-existing NAb to AAV in domestic cats, we laid a foundation for future studies leading towards gene therapy in this outbred species. Pre-existing neutralizing antibodies against AAV were assessed in 250 feline samples using an *in vitro* transduction inhibition assay. In addition, background information from the client-owned cats was analyzed in relation to the presence of NAb.

## Materials and methods

### Sample collection from client-owned and specified pathogen-free (SPF) domestic cats

In order to obtain an overview of the presence and geographic distribution of AAV NAb in cats, sera or heparin plasma samples from client-owned cats from all over Switzerland were used. From each of the 23 cantons, 10 samples were available, which resulted in 230 samples representing all of Switzerland (of note, the six half-cantons were combined into 3 full cantons). Serum or heparin plasma samples from client-owned cats were residual samples contributed by the diagnostic laboratories of the Vetsuisse Faculties in Zurich (Clinical Laboratory, 83 samples) and Bern (Zentrallabor, 59 samples), three commercial veterinary diagnostic laboratories (IDEXX Diavet Labor AG, Bäch, Switzerland, 13 samples; Labor Zentral, Geuensee, Switzerland, 11 samples and Labor am Zugersee, Switzerland, 12 samples), and various private practices in Switzerland. All samples had been obtained solely for routine diagnostic purposes during clinical presentation of the cats to the veterinarians; no additional volume was collected from any of the client-owned cats for the present study. The sole inclusion criteria were the geographic origin of the cat (area code, canton of Switzerland) and a sufficient volume of the residual sample. If available, demographic data (age, sex, reproductive status, breed of the cat) were included in the study. In addition, samples from 20 castrated male SPF cats were included. The cats originated from an approved SPF cat breeding facility and were kept in groups under optimal ethological and hygienic conditions in a barrier facility at the University of Zurich, as previously described [31]. All 20 SPF cats involved in this study were in unrelated experimental studies officially approved by the veterinary office of the Swiss Canton of Zurich (11/2011, 160/2010 and 251/2013) and conducted in accordance with Swiss laws. No additional blood collections were necessary for the present study; only residual samples were used. The ages of the SPF cats ranged from 5 months to 18 years (median age = 9.5 years).

All cat serum or plasma samples were kept in long-term storage at -80°C. Inactivated samples and dilutions were stored at -20°C for later NAb analysis.

### Cell culture

Human hepatoma cells (Huh7) or human embryonic kidney cells (HEK-293T cells) were cultivated in Advanced RPMI 1640 medium (12633012, Thermo Fisher Scientific, Fremont, USA) containing 10% fetal calf serum (FCS; 16000044, Thermo Fisher Scientific), 1% (V/V) penicillin/streptomycin (10378016, Thermo Fisher Scientific) and 2% (V/V) L-glutamine (25030081, Thermo Fisher Scientific) at 37°C in a humidified 5% CO_2_ incubator.

### AAV vectors

In the present study, AAV1, AAV2, AAV5, AAV6, AAV7, AAV8 and AAV9 vectors were used. All AAV (AAV.CMV.LacZ) vectors contained the reporter gene beta-galactosidase (LacZ), driven by a cytomegalovirus (CMV) promoter. All AAV (LacZ) vectors were constructed, purified and quantified by the Penn Vector Core at the University of Pennsylvania, as described [17].

### NAb assay

In the present study, NAb against seven different AAV serotypes were investigated by using an *in vitro* transduction inhibition assay.

The NAb assays were initially performed according to Calcedo and colleagues [17] using the Huh7 cell line, when we realized that not all AAV serotypes perform optimal within the Huh7 cells. We aimed for a low multiplicity of infection (MOI) to maximize the sensitivity of the assay. Thus, for those AAV serotypes, where the MOIs turned out to be >10’000 with the Huh7 cell line, we decided to do the assay with HEK-293T cells [32, 33]. Subsequently, the Huh7 cell line was used for the AAV serotypes AAV1, AAV2 and AAV6, whereas the HEK-293T cell line was used for the serotypes AAV5, AAV7, AAV8 and AAV9. The MOIs finally used were all in the range of 2,000–10,000 genome copy (GC)/cell with the exception of the MOI for AAV5, which was higher, namely, 20,000 GC/cell (Table 1). Several samples were compared using both Huh7 and HEK-293T cell lines and identical or similar (+/- one dilution) titers were found. This is in accordance with a paper from Wang and colleagues, where NAb titers were compared across 7 different cell lines without any significant difference [34]. The serum samples used in the present study were limited in volume. Therefore, not all samples could be run using the same cell line.

**Table 1:** List of AAV vectors, multiplicity of infection (MOI) values and cell lines used for the transduction inhibition assay.

rAAV vector	MOI (GC/cell)	Cell line
AAV1.CMV.LacZ.bGH	10,000	Huh7
AAV2.CMV.LacZ.bGH	2,000	Huh7
AAV5.CMV.LacZ.bGH	20,000	HEK-293T
AAV6.CMV.LacZ.bGH	2,000	Huh7
AAV7.CMV.LacZ.bGH	5,000	HEK-293T
AAV8.CMV.LacZ.bGH	10,000	HEK-293T
AAV9.CMV.LacZ.bGH	10,000	HEK-293T

The NAb assays were performed as described by Calcedo and colleagues [17] with few modifications. In brief, cells (Huh7 or HEK-293T) were passaged at a seeding rate of 5 × 10^4^ cells per well into 96-well plates, which were precoated with 100 μg/ml poly-D-lysine (A-003-E, Merck KGaA, Darmstadt, Germany) for HEK-293T cells, and the cells were incubated for 24 hours. Then, the cells were infected with wild-type human adenovirus 5 (HAdV5, ATCC-VR-1516, LGC Standards GmbH, Wesel, Germany) with an MOI of 50 particles per cell, and incubated for 2 hours. Cat serum or plasma samples were heat inactivated at 56°C for 35 minutes before being diluted with Advanced RPMI 1640 to final concentrations of 1:10 and 1:20. Diluted serum/plasma (100 μl) was mixed with AAV at different MOIs, dependent on the AAV serotype (Table 1), and incubated at 37°C for 1 hour. HAdV5 supernatants were removed, and AAV/cat serum mixtures were added to the cells. One hour later, 100 μl of Advanced RPMI 1640 containing 20% FCS was added to each well and incubated for 18 to 22 hours at 37°C. As controls, wells with AAV NAb-positive human serum, a non-serum control and a mock-infection (blank) were included during the transduction inhibition assay.

After cultivation, the cells were developed with beta-galactosidase (75710, Thermo Fisher Scientific) resulting in a colorimetric reaction. The supernatants were removed, and the cells were washed once with 200 μl of PBS (phosphate-buffered saline). Beta-galactosidase reagent (100 μl) was added to each well and incubated at 37°C for 30 minutes. The optical density (OD) was measured at 405 nm on a SpectraMax Plus 384 Microplate Reader (Molecular Devices, San Jose, USA). Samples that inhibited AAV transduction at a dilution of 1:20 were further diluted and tested at 1:40, 1:80, 1:160 and 1:320. The NAb titer was reported as the highest dilution that inhibited AAV.CMV.LacZ transduction by ≥50% compared to the non-serum control (as described by Calcedo et al., 2009). The limit of detection for the NAb assay was 1:10 serum or plasma dilution.

### Statistics

Statistical analyses were performed using GraphPad Prism for Windows, Version 5.03 (GraphPad Software, San Diego, CA, USA). Differences between two groups were tested for significance using the non-parametric Mann–Whitney U test for unpaired samples (*p*_MW_). Frequencies were compared using Fisher’s exact test for small numbers (*p*_F_) and the chi^2^ test (*p*_Chi2)_ otherwise. A p-value less than 0.05 was considered significant.

The linkage in seroreactivity between two different serotypes within one individual in the client-owned cats was determined according to Calcedo and colleagues [17]. In brief, the seroreactivity linkage ratio was calculated by dividing the prevalence of serotype A in the subpopulations of cats that were positive for serotype B by the prevalence of serotype A in the subpopulations of cats that were negative for serotype B. This seroreactivity linkage ratio between the two serotypes A and B (denoted by R_AB_) with a particular threshold for positivity was calculated with the formula [P_AB+_ × N_B_]/[P_B_ × P_AB-_]. P_AB+_ denotes the number of samples positive for both serotypes (A and B). N_B_ denotes the number of serum samples negative for serotype B. P_B_ denotes the number of samples positive for serotype B and P_AB-_ denotes the number of samples positive for serotype A and negative for serotype B. A ratio >1 suggests a positive linkage (i.e., seroreactivity to one virus is associated with an increased frequency of seroreactivity to another virus), whereas a ratio <1 indicates a negative association (i.e., seroreactivity to one virus reduces reactivity to a second virus) [17].

The maps were created using Quantum Geographic Information system (QGIS), Version 3.2.0.

## Results

### Sample characteristics of client-owned cats

In this study, samples from 230 cats from all over Switzerland were included. Of these cats, 47% were specified male (n = 109), 40% were specified female (n = 92) and 13% were of unspecified sex (n = 29). The majority of the client-owned cats were European Shorthairs (54%, n = 125); some cats were Birmans (3%; n = 8), British Shorthairs (3%, n = 7) or other breeds, such as Siamese, Bengals, Maine Coons, Ocicats, and others (18%; n = 41); for 21% (n = 49) of the cats, no information on breed was available. The ages of the cats ranged from 6 weeks to 21 years (median age = 9.5 years), and 3% were of unspecified age (n = 8).

### Seroprevalence of AAV NAb in client-owned cats

**Fig 1:**
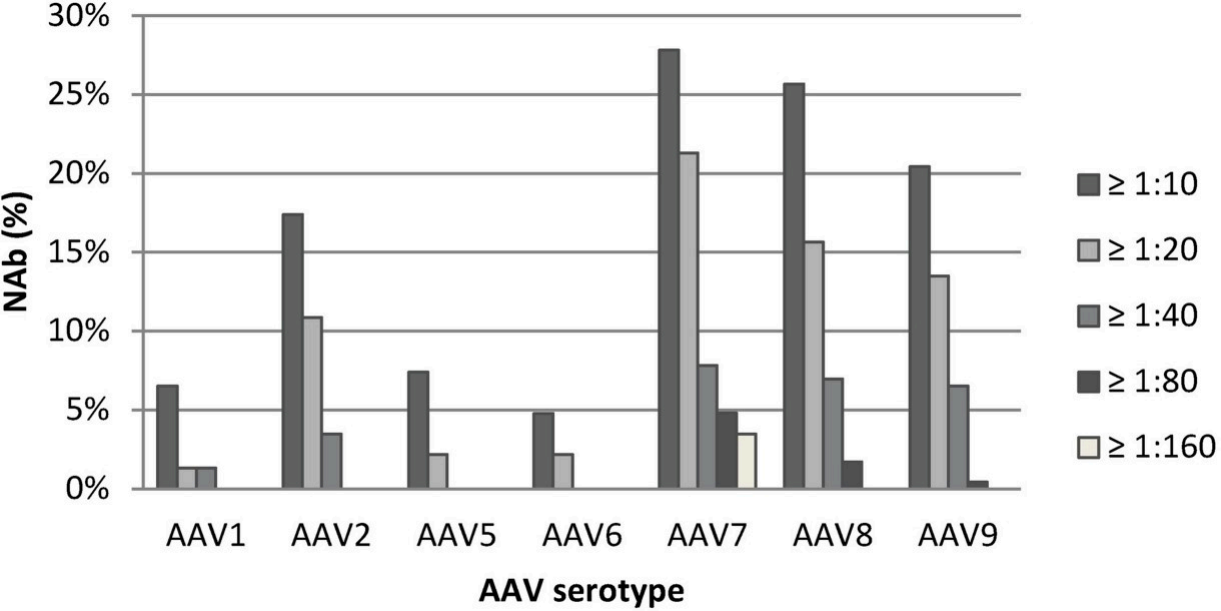
Prevalence of pre-existing NAb against AAV serotypes in client-owned domestic cats from Switzerland depicted according to titers. The percentage of NAb against different AAV serotypes is shown for serum dilutions of ≥1:10; ≥1:20, ≥1:40, ≥1:80 and ≥1:160. NAb against AAV7, AAV8 and AAV9 were the most prevalent; NAb against AAV1, AAV5 and AAV6 were the least prevalent.

**Fig 2:**
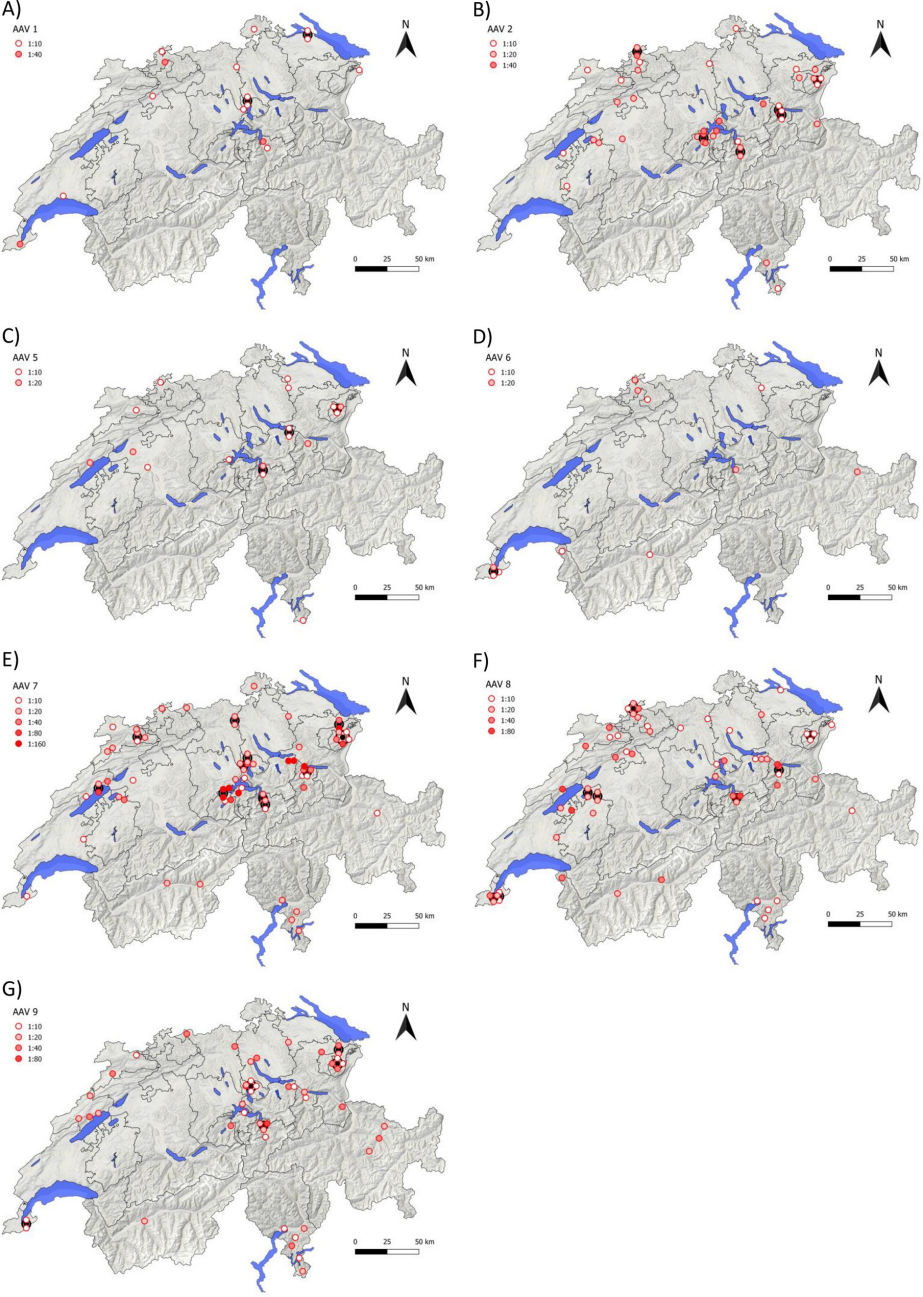
Geographic origin of cats with NAb against AAV in Switzerland. NAb against A) AAV1, B) AAV2, C) AAV5, D) AAV6, E) AAV7, F) AAV8, and G) AAV9 are shown. Dots represent samples with positive NAb against an AAV serotype. Higher color intensity refers to higher positive NAb titers, with 1:160 being the darkest. Black dots represent geographic locations where more than one sample with a positive NAb was found. In this case, all positive samples are clustered around the black dot.

Samples from the 230 client-owned cats and 20 SPF cats were analyzed for the presence of NAb against AAV1, AAV2, AAV5, AAV6, AAV7, AAV8 and AAV9. NAb titers from all client-owned cats are summarized in Fig 1; the geographic origin of the samples with NAb is depicted in Fig 2 and listed in S1 and S2 Tables categorized into the different cantons and regions. Overall, 122 client-owned cats showed positive neutralizing activity (53%), while in 108 cats no NAb were detected against any of the tested AAV serotypes (47%). The majority of the client-owned cats with neutralizing activity (75 of 122 cats; 61%) had NAb against more than one of the tested AAV serotypes: 38 cats had NAb against two serotypes, 25 cats against three serotypes, seven cats against four serotypes, four cats against five serotypes and one cat against all seven AAV serotypes investigated.

There were significant differences in the prevalence of NAb against the various AAV serotypes (p_Chi2_ < 0.0001; Fig 1). When titers ≥1:10 were included, NAb against AAV6 were the least common (5%) and NAb against AAV7 were the most common (28%). NAb against AAV1, AAV5 and AAV6 were significantly less prevalent than NAb against AAV7, AAV8 or AAV9 (*p*_F_ < 0.0001). The prevalence of NAb against AAV2 was intermediate, at 17%. When only titers ≥1:20 or ≥1:40 were analyzed, the general NAb distribution remained similar to what was found when all titers ≥1:10 were included (Fig 1). Again, high NAb titers against AAV7, AAV8 and AAV9 were significantly more frequently found than NAb to AAV1, AAV5 or AAV6 (*p*_F_ < 0.0001). The highest NAb titers were found against AAV7 (≥1:160), while AAV5 and AAV6 reached titers of only ≥1:20 (Fig 1).

Significant geographic differences among the different cantons of Switzerland were found for the presence of NAb against AAV2, AAV7 and AAV9 (*p*_Chi2_ < 0.0001 for AAV2 and AAV7; *p*_Chi2_ < 0.0039 for AAV9; Fig 2). In particular, the cantons of Unterwalden, Zug and Appenzell showed high prevalence (e.g., 70%, 60%, and 60%, respectively, for AAV7), whereas in the cantons of Zurich, Bern and Lucerne, rather low prevalence was found (e.g., 0%, 10%, and 20%, respectively, for AAV7; S1 Table).

### Age, breed and sex of the client-owned cats and presence of NAb against AAV

When titers ≥1:10 were considered, the age of the cats with NAb to AAVs was not significantly different from the age of the cats without detectable NAb to AAVs; this was true for NAb against each AAV serotype as well as the combined AAV serotypes (S1 Fig). At higher titers, significant differences were found for NAb against AAV2 (titers ≥1:20, *p*_MW_ = 0.0350 [S1J Fig] and ≥1:40, *p*_MW_ = 0.0140 [S1R Fig]) and AAV7 (titers ≥1:40, *p*_MW_ = 0.0194 [S1S Fig]; ≥1:80, *p*_MW_ = 0.0354 and ≥1:160, *p*_MW_ = 0.0121 [S1W and S1AA Fig, respectively]), where cats with NAb were significantly younger than cats with low or no titers.

No significant difference in the presence of NAb to AAV serotypes (titers ≥1:10, ≥1:20 and ≥1:40) could be detected between purebred cats (all breeds combined) and non-purebred cats (European Shorthairs and mixed-breeds); again, this was true for NAb against each of the tested AAV serotypes (S2 Fig), with the exception of NAb titers ≥1:40 against AAV2 (*p*_F_ = 0.0038; S2R Fig) and ≥1:160 against AAV7 (*p*_F_ = 0.0115, S2AK Fig). In these cases, purebred cats had a significantly higher NAb frequency than non-purebred cats.

Moreover, there was no significant difference in the prevalence of NAb to AAV between male and female cats (≥1:10, ≥1:20, ≥1:40, ≥1:80 and ≥1:160) when NAb against each serotype were analyzed separately (S3 Fig). However, when NAb to all AAV were analyzed together, female cats had a significantly higher frequency of NAb titers ≥1:40 than male cats had (*p*_F_ = 0.0046; S3X Fig).

### Seroreactivity linkage analyses

To evaluate whether there was a linkage in the presence of NAb against two different AAVs within one individual, a seroreactivity linkage analysis was performed. Most of the comparisons showed modest, as defined by Calcedo and colleagues [[17]; ratios: 1.8–3.0] or no correlations (Table 2). Ratios higher than 3 were found between various AAV serotypes (Table 2). The strongest positive correlation was observed between AAV6 and AAV8 (ratio 13.0).

**Table 2:** Linkage of seroreactivity (R_AB_) between AAV serotypes in client-owned cats in Switzerland (NAb titer: 1:10).

	Seroreactivity linkage ratio (R_AB_)						
	AAV1	AAV2	AAV5	AAV6	AAV7	AAV8	AAV9
**AAV1**	NA	1.2	0.9	**7.2**	1.3	2.5	1.0
**AAV2**	1.2	NA	2.3	1.6	**3.9**	2.1	2.9
**AAV5**	0.9	2.6	NA	1.2	**3.7**	**4.1**	2.7
**AAV6**	**8.2**	1.8	1.3	NA	1.0	**13.0**	2.2
**AAV7**	1.2	2.9	2.3	1.0	NA	2.4	**3.4**
**AAV8**	1.9	1.9	2.6	**3.6**	2.5	NA	2.5
**AAV9**	1.0	2.7	2.2	1.9	**4.6**	2.8	NA

NA: Not applicable. A ratio of >1 suggests a positive linkage (i.e., presence of NAb to one AAV is associated with an increased frequency of seroreactivity to another AAV); a ratio <1 indicates a negative association. Bold numbers represent linkage ratios >3. More details on the calculations can be found in Materials and Methods.

### Seroprevalence of AAV NAb in SPF cats

SPF cats showed no antibodies to AAVs with the exception of one cat [35], which had NAb against AAV2 and AAV7 with a titer of ≥1:10. Thus, in SPF cats, the 5% prevalence of NAb against AAV2 and AAV7 is lower than the prevalence of such NAb in client-owned cats, which measured 17% and 28%, respectively (not significant for AAV2, *p*_F_ = 0.2131, but statistically significant for AAV7, *p*_F_ = 0.0305).

## Discussion

The present study focused on investigating NAb against seven different AAV serotypes in the domestic cat population of Switzerland. This is the first study to use an *in vitro* transduction inhibition assay to test for biologically active NAb to AAVs in cats, as well as the first investigation of AAV seroprevalence in a large number of cats (n = 250). There has been only one previous study on the subject in cats; that study was smaller and examined total antibodies to AAVs in cats using an ELISA technique [36].

In numerous studies cats are serving as animal models for human diseases, examples include AIDS, diabetes, cancer and Alzheimer’s disease [24, 37–39]. Cats are, in contrast to laboratory mice, an outbred species, have a longer lifespan, are bigger and show many monogenetic disorders like humans. Thus, studies in cats using gene therapy to treat diseases, such as the mucopolysaccharidosis type VI (MPS VI), have great potential. Treated cats showed long-term improvements including clearance of glycosaminoglycans, reduction of heart valve thickness, improvement of long bone length and spontaneous mobility [40]. However, one study showed that pre-existing immunity even at low levels (1:5/1:10 to undetectable) had a negative impact on AAV8-mediated liver gene transfer in MPS VI cats, severely limiting therapeutic efficacy [30].

Gene therapy has also been suggested in domestic cats, e.g. to permanently sterilize the animals for population control and to avoid euthanasia of healthy adult cats [36]. Based on estimations, there are approximately 1.6 million cats living in Switzerland in 2018 (https://de.statista.com). Of these cats, approximately 100,000–300,000 cats were stray cats (http://www.shkr.ch). Worldwide, estimates of cat numbers are around 600 million (http://www.ecology.com), including a significant number of stray cats. Thus, the benefit of a one-time gene therapy in comparison with a surgery in potentially millions of cats worldwide would be substantial. An environment matched cat population would significantly improve animal welfare as well as reduce the cat’s effect on the ecological balance (potential threat to reptiles, birds and small mammals) and their potential role in serving as vectors for human diseases [41].Thus, an inexpensive, simple method to induce permanent infertility leading to a stable cat population is highly desirable.

In the last few decades, several methods have been developed to detect antibodies against AAV. In the 1970s, measurements of total antibodies to AAV1 and AAV2 –the available serotypes at the time–were performed by ELISA and Western blot [42]. In order to determine serotype-specific NAb and not only binding Ab, *in vitro* transduction inhibition assays and *in vivo* methods were developed. The *in vivo* assay uses mice, which are injected with the test serum sample prior to vector delivery [43, 44]. The vector expresses a secreted reporter gene whose expression is compared to the level in control mice injected with only the AAV vector. This assay is highly sensitive; however, it is also time consuming, more expensive than *in vitro* methods and reliant on animal experiments, which goes against the 3Rs principle for animal welfare. In contrast, the *in vitro* transduction inhibition assay measures a reporter gene after transduction with an AAV vector preincubated with the test serum [17, 45, 46]. Thus, this assay does not involve experimental animals, has a high throughput capacity, and is shown to have a good correlation with *in vivo* transduction in macaques (Calcedo and Wilson 2013). Therefore, the *in vitro* transduction inhibition assay for the determination of NAb to AAVs is currently the most widely used method to evaluate clinical samples.

Initially, it was assumed that only nonhuman primates and humans have pre-existing NAb against AAV, since no NAb to AAV were detected in rats [47]. Subsequently, however, NAb to AAV were detected in other mammalian species, such as the mouse, rabbit, dog and pig (see Table 3 for an overview) [33, 48]. It became evident that the prevalence of NAb depends on both the AAV serotype and the host species [33, 48]. In the present study, NAb to seven AAV serotypes were determined in the Swiss cat population. However, it needs to be mentioned that at present we do not know whether NAb detected in cats against the various rAAVs were raised against human AAVs or are cross-reactive NAb to feline wild-type AAVs. Both explanations should be considered: Close contact with humans, where AAV infections appear to be endemic, could result in cross-species infection, leading to cats showing NAb against specific human AAV serotypes. Transmission between hosts has been suggested based on the phylogenetic relationship of AAV isolates between humans and non-human primates [49], as well as humans and ruminants [50]. In addition, it has been shown that pigs obtained from privately owned farms were more frequently AAV infected than pigs from slaughterhouses [51]. This could suggest that close contact of farmed pigs to humans provides an environment for zoonotic transmission. Thus, the close contact between cats and humans might lead to cats showing NAb against specific human AAV serotypes. Alternatively, previous infection with a potential feline wild-type AAV should to be considered as source of cross-reactive NAb against human AAVs.Indeed, using an AAV2-ITR specific qPCR assay [52], 54 Swiss field cats were tested for the presence of AAV2. Four (7%) of these samples tested low positive for rAAV2 (Ct values >36). Due to the high Ct values (low loads), no sequencing for confirmation of the PCR results was possible. Thus, future investigations of the potential presence of feline or human AAVs in domestic cats will be interesting.

**Table 3:** Overview of the prevalence (in %) of NAb to AAV in various animal species.

	AAV1	AAV2	AAV5	AAV6	AAV7	AAV8	AAV9
**Pig**^a^*	47	30	100	6	NA	35	NA
**Cat**^**b**^	7	17	7	5	28	26	20
**Dog**^a^	100	0	0	100	NA	0	NA
**Horse**^a^*	0	8	100	8	NA	0	NA
**Nonhuman primate**^**c**^			17*		≤100	≤100	≤100*

^a^[48]

^b^present study

^c^[53, 54]

* small number of animals in the study, n < 20.

NAb were detected against all seven serotypes, with the highest prevalence of NAb to AAV7, AAV8 and AAV9 (20–28%). This prevalence is lower than what was reported in 35 client-owned cats from the Animal Health Diagnostic Center of Cornell University (30–80%) [36]. However, as mentioned above, not NAb but total anti-AAV-capsid antibodies were measured in that study, a fact that may readily explain the discrepancies in prevalence between the two studies. Our results partially mirror the situation in nonhuman primates, including macaques, squirrel monkeys, chimpanzees and baboons [53, 54], where AAV7, AAV8 and AAV9 were also the serotypes with the highest prevalence of NAb. However, up to 100% of the nonhuman primates were positive for NAb to AAV7, AAV8 and AAV9 [53, 54], while in our study the NAb prevalence never reached 30% for any serotype tested (Table 3). In horses and pigs, NAb to AAV5 were the most prevalent (100%); in dogs, NAb to AAV1 and AAV6 were found in 100% of the samples tested [48, 55]; and in humans, NAb to AAV2 were found frequently (30–72%) [17, 42, 45, 56].

Co-occurrence of NAb to another AAV serotype was found in 61% of cases in the present study. Previous studies have determined an even higher co-prevalence of up to 91% in humans [56].

Regarding neutralization titers, titers of up to 1:12,800 were found against AAV2 in humans [56]. Generally, lower titers were detected in cats, with AAV7 having the highest NAb titers (1:160), followed by AAV8 and AAV9 (1:80; Fig 1). Findings in horses showed the highest titers for NAb to AAV5, with titers of up to 1:80 [48]; in dogs, to AAV1 and AAV6, with titers of up to 1:640 and 1:1024, respectively [33, 48]; and in pigs, to AAV1, AAV2, AAV5 and AAV8, with titers of up to 1:160 [33, 48]. Thus, titers as high as the ones described in humans have not been found in large animals. However, titers seem to vary substantially between different species. Overall, the prevalence of NAb to AAVs in the cat population investigated in the current study were considerably lower than what has been reported from other species; on the other hand, in one cat NAb were found against all the AAVs investigated and some cats had NAb against several of the AAV serotypes.

The samples included in the present study were collected from cats during veterinary consultation. Most of the cats, therefore, were probably clinically ill, or they were healthy and being presented for vaccination, castration, or other procedures. Thus, our study gives the prevalence of NAb to AAVs in cats presented to veterinarians. This population may at least partially represent the cats that are candidates for a future gene therapy approach. The samples investigated in this study were residual samples, collected in the clinics for unrelated reasons. The available volume, which is usually quite limited for feline samples, defined the minimal dilution investigated in the neutralization assay (which was 1:10). Dilutions of 1:2 or 1:5 were not feasible. Earlier publications state that AAV8 NAb titers of 1:20 were enough to completely block transduction in passively immunized mice [48] and that AAV8 NAb titers greater than 1:10 were enough to significantly reduce AAV8 transduction of the liver in monkeys [57].

Despite the rather small area of Switzerland, significant geographic differences in the prevalence of NAb were found among the distinct cantons. In particular, the cantons of Unterwalden, Zug and Appenzell showed high prevalence, whereas the cantons of Zurich, Bern and Lucerne had rather low prevalence. Regional differences had already been described, with significantly higher frequencies of NAbs to all AAV serotypes in humans from Africa than in those from other continents [17], as well as differences in the prevalence of NAb against AAV1 in two separate regions of China [58]. Both studies stated that significantly higher NAb against AAV were detected in developing than developed regions, and it was speculated that living conditions, population density, hygienic conditions or MHC background could be involved in this phenomenon [17, 58]. In fact, in the present study, cats from typical urban regions showed the lowest NAb prevalence (Zurich, Bern and Lucerne), whereas cats from rural regions (Unterwalden, Appenzell) revealed higher NAb prevalence. It might be speculated that, indeed, cats from rural regions have more outdoor access and are more likely to live in multi-cat households, resulting in more antigen exposure than typical urban cats. Interestingly, NAb frequency against AAV2 was significantly higher in purebred cats than non-purebred cats (titers ≥1:40 against AAV2; *p*_F_ = 0.0038; S2R Fig). In contrast to non-purebred cats, purebred cats might be more likely restricted to indoors and have closer contact to humans, where AAV2 infections seem to be quite common. However, in order to solidify either hypothesis more cats and additional information, such as outdoor access, etc., would need to be investigated.

Although no significant link between the presence of NAb against single AAV serotypes and the sex of the cat could be found, combined NAb to AAV at a titer of ≥1:40 showed significantly higher NAb prevalence in females than in males (*p*_F_ = 0.0046; S3X Fig). Earlier studies showed that the immune system is broadly affected by the sex of the animal [59, 60]. Intact female cats showed a lower rate of apoptotic lymphocytes after overnight culture than castrated cats, and estradiol protected peripheral lymphocytes from apoptosis after stimulation in female cats [61]. B lymphocytes produce antibodies; if apoptosis of B lymphocytes is lower in female than male cats, this may contribute to higher NAb in females. However, only information on sex and not on castration status was available from the cats in the present study.

The linkage of seropositivity for distinct AAV serotypes was not prominent for the majority of serotypes investigated (Table 2). Most seroreactivity ratios showed modest correlations, as defined by Calcedo and colleagues ([17]; ratios: 1.8–3.0). The strongest correlation of seroreactivity was observed between AAV6 and AAV8 (ratio of 13.0), meaning that most cats seropositive for AAV6 were also seropositive for AAV8. However, it must be noted that ratios higher than the present ratio of 13.0 have been described for human samples, with values >100 [17]. Previous publications from studies in humans suggest that cross-reactive antibodies to AAV5 and AAV6 were generated after exposure to wild-type AAV2 [56]. In the present study, such cross-reactivity of NAb to AAV2 and AAV5 or AAV6 was not observed. Due to the high similarity of capsids of AAV1 and AAV6 in humans (99.2% homology, [62]), one would expect a high linkage of seropositivity. Indeed, a rather strong linkage of seroreactivity was also observed here with ratios of 7.23 and 8.19 (Table 2). Most serotypes investigated showed absence of substantial linkage of seropositivity, which is in accordance with several studies investigating repeated AAV administration in mice [63, 64]. In addition, it was shown that the various AAV serotypes differ in their immunogenic potential. Thus, at present we can only speculate whether the low levels of linkage of seropositivity are due to sequence variations between human AAVs and potential feline AAVs and/or differences in the immunogenicity of the AAVs and the immune reaction of the cat. It has been considered that co-occurrence of NAb might be either the result of multiple infections or co-infections with several AAV within the same individual [56].

As expected, most SPF cats did not have detectable levels of NAb, with the exception of one cat, which had low titers of NAb against AAV2 and AAV7. This cat was infected with FeLV-A/Glasgow-1 in an unrelated study [35] and had developed acute leukemia at the time of euthanasia when the serum was collected for this study. In contrast to a study by Dissen and colleagues [36], where recently vaccinated SPF cats showed cross-reactive NAb against AAV6, the SPF cats in the present study had never been vaccinated to the best of our knowledge. In agreement with this, no antibodies were detected for parvo-, herpes-, or calicivirus in the serum of the AAV NAb-positive cat (S3 Table and S1 File). Thus, it remains unclear at present how the cat acquired the AAV NAb.

Several studies have shown that pre-existing NAb against AAV present a significant hurdle for gene therapy [5, 13, 14]. In the Calcium Upregulation by Percutaneous Administration of Gene Therapy in Cardiac Disease (CUPID) study, almost 50% of patients had to be excluded from therapy due to NAb titers of >1:2 [19]. In the first hemophilia B clinical trial, NAb titers of 1:17 completely blocked detectable levels of factor IX transgene expression, while another subject with a titer of 1:2 developed only low levels of expression [5]. Thus, even low titers of pre-existing NAb may significantly neutralize high doses of vector. However, although anti-AAV immune responses can result in loss of transgene expression, it is primarily the efficiency of the gene therapy and not its safety that is at stake [15]. Moreover, the presence of NAb might not influence all gene therapy studies due to the route of administration or the large vector doses per kg body weight used. Interestingly, the presence of NAb against AAV does not impede transduction when administered by the intraparenchymal route, into the subretinal space [65] or into the cerebral ventricle [66]. Little to no capsid- or transgene-specific immune response could be detected in serum or PBMCs after gene therapy to immune-privileged compartments of the body such as the eye or central nervous system [67]. Probably the immunologic unresponsiveness of the brain and eye is dependent on the vector dose; thus, future studies will define the upper limit for the vector dose [67].

Despite the broad usage of AAV vectors in clinical trials, pre-existing antibodies always remain a barrier for the therapy efficacy. To solve this issue, an alternative strategy uses the AAV hybrid strain AAV-DJ to escape NAb *in vivo*. This strain was derived from 8 wild serotypes of AAV (AAV2, AAV4, AAV5, AAV8, AAV9, avian, bovine, caprine) by DNA family shuffling technology [68]. During screening, the AAV-DJ strain was chosen as an immune-escaping strain from the quasispecies library incubated with pools of polyclonal antibodies against each AAV serotype. Since each wild serotype has its own tropism, the chimera inherited all the parental tropisms and showed almost equipotent infection efficiency throughout the organs in mice [68]. In a preliminary experiment, we selected 20 samples of the current study from client-owned domestic cats that had NAb against several AAV serotypes. The majority of the samples (17/20) were not capable of neutralizing AAV-DJ (titer <1:10). The exceptions were one sample with very broad neutralizing capacity (Uri #78), which had shown NAb against all investigated serotypes, and two samples (Schwyz #83 and Glarus #114) that had neutralized five or four of the seven investigated AAV serotypes, respectively (S4 Fig and S1 File). Thus, these preliminary results indicate that in domestic cats, lower levels of pre-existing NAb to AAV-DJ can be expected compared to wild-type AAV serotypes. Future studies in the cat may thus also concentrate on using AAV-DJ.

## Conclusions

Compared with the prevalence of NAb against AAV in humans, the prevalence of NAb in cats was reduced, ranging from 5%–28%. A low prevalence of NAb against AAV in cats supports its applicability for gene therapy. However, due to the potential hindrance of NAb during gene therapy and the currently rather high effort necessary for gene therapy, prescreening of the individual animals for the presence of NAb to the AAV serotype in question may be advisable.

## Supporting information

S1 Table Pre-existing prevalence of NAb against various AAV serotypes in domestic cats shown for each canton of Switzerland. (DOCX)

S2 Table Pre-existing NAb against various AAV serotypes in domestic cats, divided according to the regions of Switzerland. (DOCX)

S3 Table Immunofluorescence assay (IFA)-results of the AAV NAb-positive SPF cat (QLK1). (DOCX)

S1 Fig Comparison of the ages of cats with and without NAb against AAV. Shown here are comparisons of the ages of cats with and without NAb against AAV1, AAV2, AAV5, AAV6, AAV7, AAV8, AAV9 and all AAV serotypes combined, for the titers ≥1:10 (A-H), ≥1:20 (I-P), ≥1:40 (Q-V), ≥1:80 (W-Z) and ≥1:160 (AA). Samples were considered positive if the respective serum dilution inhibited transduction by ≥50%. A comparison of the ages of cats with and without NAb against each AAV serotype or all combined were analyzed using the Mann–Whitney U test (p_*MW*_). A p-value less than 0.05 was considered significant. The data are shown as box plots; the boxes extend from the 25th to the 75th percentile. The horizontal line represents the median, and the whiskers extend from the smallest to the largest value.
(PDF)

S2 Fig Cat breeds and the presence of NAb. Depicted here are the cats grouped as purebred (including Birmans, Ocicats, Siamese and others) or non-purebred (European Shorthairs and mixed breeds) and as having or lacking NAb against AAV1, AAV2, AAV5, AAV6, AAV7, AAV8, AAV9 and all AAV serotypes combined, for the titers ≥1:10 (A-H), ≥1:20 (I-P), ≥1:40 (Q-X), ≥1:80 (Y-AF) and ≥1:160 (AG-AN). Frequencies were compared using Fisher’s exact test for small numbers (*p*_F_). A p-value less than 0.05 was considered significant. No statistically significant difference could be detected when the prevalence of NAb against various AAV serotypes was compared between European Shorthairs (and mixed breeds) and other cat breeds (*p*_F_ > 0.05) at a titer of ≥1:10. The numbers within the columns represent the numbers of cats included in the analysis. A total of 56 purebred and 125 non-purebred cats were included. Samples were considered positive if the respective serum dilutions inhibited transduction by ≥50%.
(PDF)

S3 Fig The sexes of the cats and the presence of NAb. Cats are grouped by sex (male and female cats) and the presence or absence of NAb against AAV1, AAV2, AAV5, AAV6, AAV7, AAV8, AAV9 and all AAV serotypes combined, for the titers ≥1:10 (A-H), ≥1:20 (I-P), ≥1:40 (Q-X), ≥1:80 (Y-AF) and ≥1:160 (AG-AN). Frequencies were compared using Fisher’s exact test for small numbers (*p*_F_). A p-value less than 0.05 was considered significant. A statistically significant difference could be detected between sexes when the prevalence of NAb against all AAV serotypes combined was compared at a titer of ≥1:40 (*p*_F_ > 0.0046, n = 201; 109 male and 92 female). Samples were considered positive if the respective serum dilutions inhibited *in vitro* transduction by ≥50%.
(PDF)

S4 Fig Presence of NAb against AAV-DJ in selected client-owned domestic cats. A transduction inhibition assay was performed in order to determine the presence of NAb against AAV-DJ-EGFP. Twenty cat serum samples previously shown to have NAb against other AAV serotypes were chosen. Two different serum dilutions were tested for each sample: 1:10 and 1:20. Controls included an AAV-NAb-positive human serum (H2 1:20), a non-serum control (AAV-DJ) and cells only (mock-infection without a virus; Neg.). Descriptions within each picture refer to the specific serum/plasma samples. The bar represents 400 nm.
(PDF)

S1 File Supporting materials and methods. (DOCX)
