# Supplementary material for: Pre-existing antibodies to candidate gene therapy vectors (adeno-associated vector serotypes) in domestic cats
Source: PLoS One. 2019 Mar 21;14(3):e0212811. doi: 10.1371/journal.pone.0212811 (PMC6428272; doi:10.1371/journal.pone.0212811)
Supplement: S1 Table — (DOCX) [file pone.0212811.s001.docx]

**S1 Table** Pre-existing prevalence (in % of positive samples within each canton) of NAb with titers ≥1:10 against various AAV serotypes in domestic cats shown for each canton of Switzerland (CH).

|  | **AAV1** | **AAV2** | **AAV5** | **AAV6** | **AAV7** | **AAV8** | **AAV9** |
| --- | --- | --- | --- | --- | --- | --- | --- |
| **Zurich** | 0 | 0 | 0 | 0 | 0 | 10 | 20 |
| **Bern** | 0 | 10 | 20 | 0 | 10 | 0 | 0 |
| **Lucerne** | 0 | 10 | 0 | 0 | 20 | 10 | 10 |
| **Uri** | 20 | 30 | 20 | 10 | 40 | 30 | 40 |
| **Schwyz** | 0 | 10 | 20 | 0 | 20 | 30 | 20 |
| **Unterwalden** | 0 | 70 | 10 | 0 | 70 | 0 | 20 |
| **Glarus** | 0 | 40 | 10 | 0 | 60 | 40 | 20 |
| **Zug** | 30 | 0 | 0 | 0 | 60 | 20 | 50 |
| **Fribourg** | 0 | 40 | 0 | 0 | 20 | 60 | 0 |
| **Solothurn** | 20 | 40 | 0 | 10 | 0 | 40 | 0 |
| **Basel** | 10 | 20 | 10 | 20 | 10 | 60 | 0 |
| **Schaffhausen** | 10 | 10 | 0 | 0 | 10 | 0 | 0 |
| **Appenzell** | 0 | 50 | 30 | 0 | 60 | 30 | 50 |
| **St. Gallen** | 10 | 20 | 0 | 0 | 20 | 20 | 30 |
| **Grisons** | 0 | 0 | 0 | 10 | 10 | 10 | 30 |
| **Aargau** | 10 | 10 | 0 | 0 | 30 | 20 | 20 |
| **Thurgau** | 20 | 0 | 20 | 10 | 10 | 20 | 10 |
| **Ticino** | 0 | 20 | 10 | 0 | 40 | 40 | 60 |
| **Vaud** | 10 | 0 | 0 | 0 | 10 | 20 | 0 |
| **Valais** | 0 | 0 | 0 | 20 | 20 | 30 | 10 |
| **Neuchâtel** | 0 | 0 | 10 | 0 | 40 | 10 | 40 |
| **Geneva** | 10 | 0 | 0 | 30 | 10 | 50 | 20 |
| **Jura** | 0 | 20 | 10 | 0 | 70 | 40 | 20 |
