# Supplementary material for: Pre-existing antibodies to candidate gene therapy vectors (adeno-associated vector serotypes) in domestic cats
Source: PLoS One. 2019 Mar 21;14(3):e0212811. doi: 10.1371/journal.pone.0212811 (PMC6428272; doi:10.1371/journal.pone.0212811)
Supplement: S2 Table — (DOCX) [file pone.0212811.s002.docx]

**S2 Table** Pre-existing NAb with titers ≥1:10 against various AAV serotypes in domestic cats, shown as percentages and divided according to the regions of Switzerland (CH) as defined by the Federal Statistical Office (BFS).

| **Region of CH** | **AAV1** | **AAV2** | **AAV5** | **AAV6** | **AAV7** | **AAV8** | **AAV9** |
| --- | --- | --- | --- | --- | --- | --- | --- |
| **Lake Geneva^1^** | 7 | 0 | 0 | 17 | 13 | 33 | 10 |
| **Central Plateau^2^** | 4 | 22 | 8 | 2 | 28 | 30 | 12 |
| **Northwestern CH^3^** | 10 | 15 | 5 | 10 | 20 | 40 | 10 |
| **Zurich** | 0 | 0 | 0 | 0 | 0 | 10 | 20 |
| **Eastern CH^4^** | 7 | 20 | 10 | 3 | 28 | 20 | 23 |
| **Central CH^5^** | 10 | 24 | 10 | 2 | 42 | 18 | 28 |
| **Ticino** | 0 | 20 | 10 | 0 | 40 | 40 | 60 |
| **Total CH** | 7 | 17 | 7 | 5 | 28 | 26 | 20 |

^1^Cantons of Geneva, Vaud and Valais; ^2^Cantons of Bern, Solothurn, Fribourg, Neuchâtel and Jura; ^3^Cantons of Basel and Aargau; ^4^Cantons of St. Gallen, Appenzell, Glarus, Schaffhausen, Grisons, Thurgau; ^5^Cantons of Uri, Schwyz, Unterwalden, Lucerne, Zug.
