# Supplementary material for: Pre-existing antibodies to candidate gene therapy vectors (adeno-associated vector serotypes) in domestic cats
Source: PLoS One. 2019 Mar 21;14(3):e0212811. doi: 10.1371/journal.pone.0212811 (PMC6428272; doi:10.1371/journal.pone.0212811)
Supplement: S3 Table — (DOCX) [file pone.0212811.s003.docx]

**S3 Table** **Immunofluorescence assay (IFA)-results of the** **AAV NAb-positive cat (QLK1).** FHV-1: Feline herpes virus 1; FCV: Feline calicivirus; FPV: Feline parvovirus. All titers were considered negative as they were lower than 1:20.

| **Antigen** | **IFA** | **Titer** |  |  |  |  |  |
| --- | --- | --- | --- | --- | --- | --- | --- |
| **FHV-1** | negative | <1:20 |  |  |  |  |  |
| **FCV** | negative | <1:20 |  |  |  |  |  |
| **FPV** | negative | <1:20 |  |  |  |  |  |
