# Supplementary figures and images for: Pre-existing antibodies to candidate gene therapy vectors (adeno-associated vector serotypes) in domestic cats
Source: PLoS One. 2019 Mar 21;14(3):e0212811. doi: 10.1371/journal.pone.0212811 (PMC6428272; doi:10.1371/journal.pone.0212811)

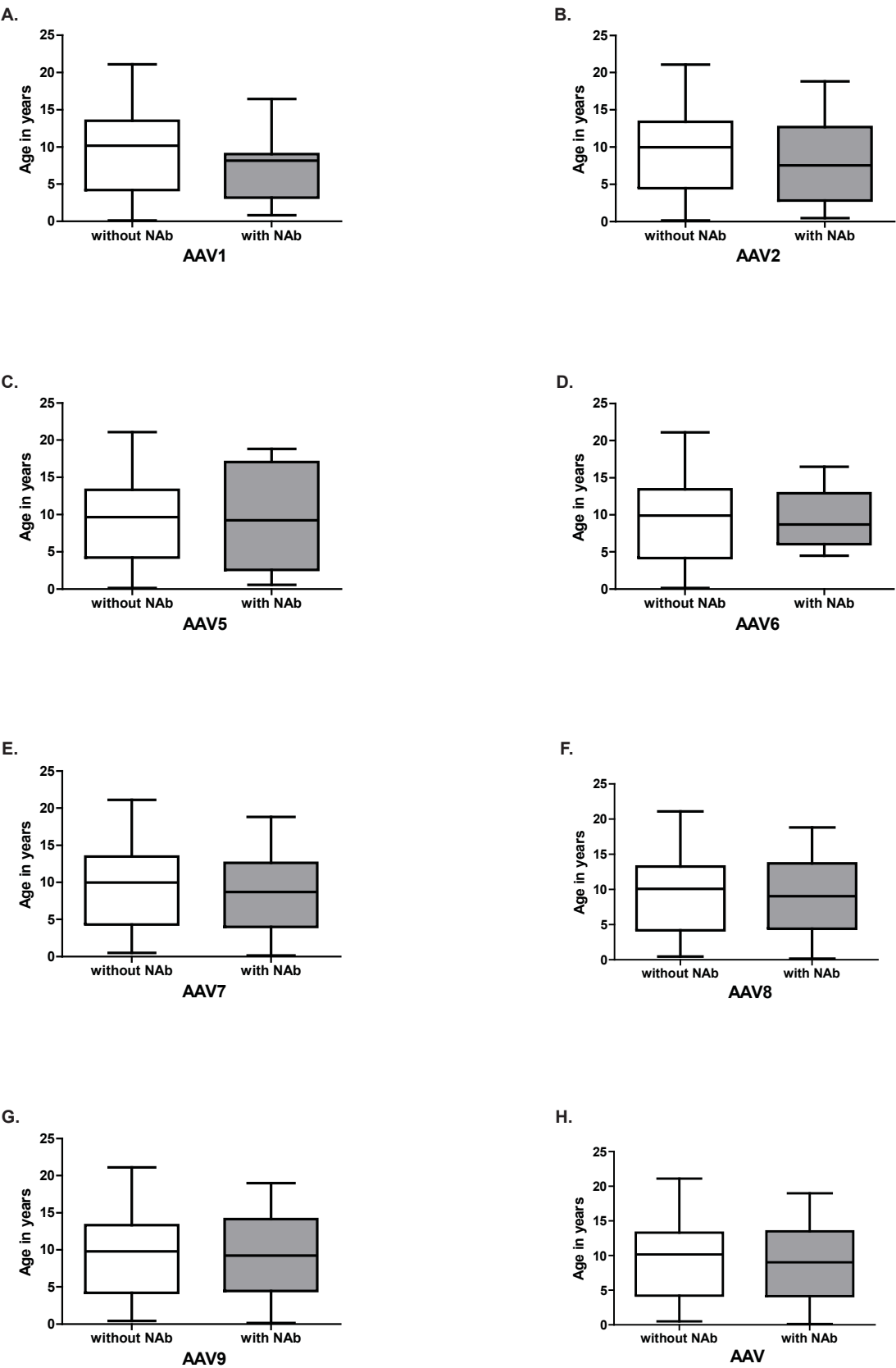

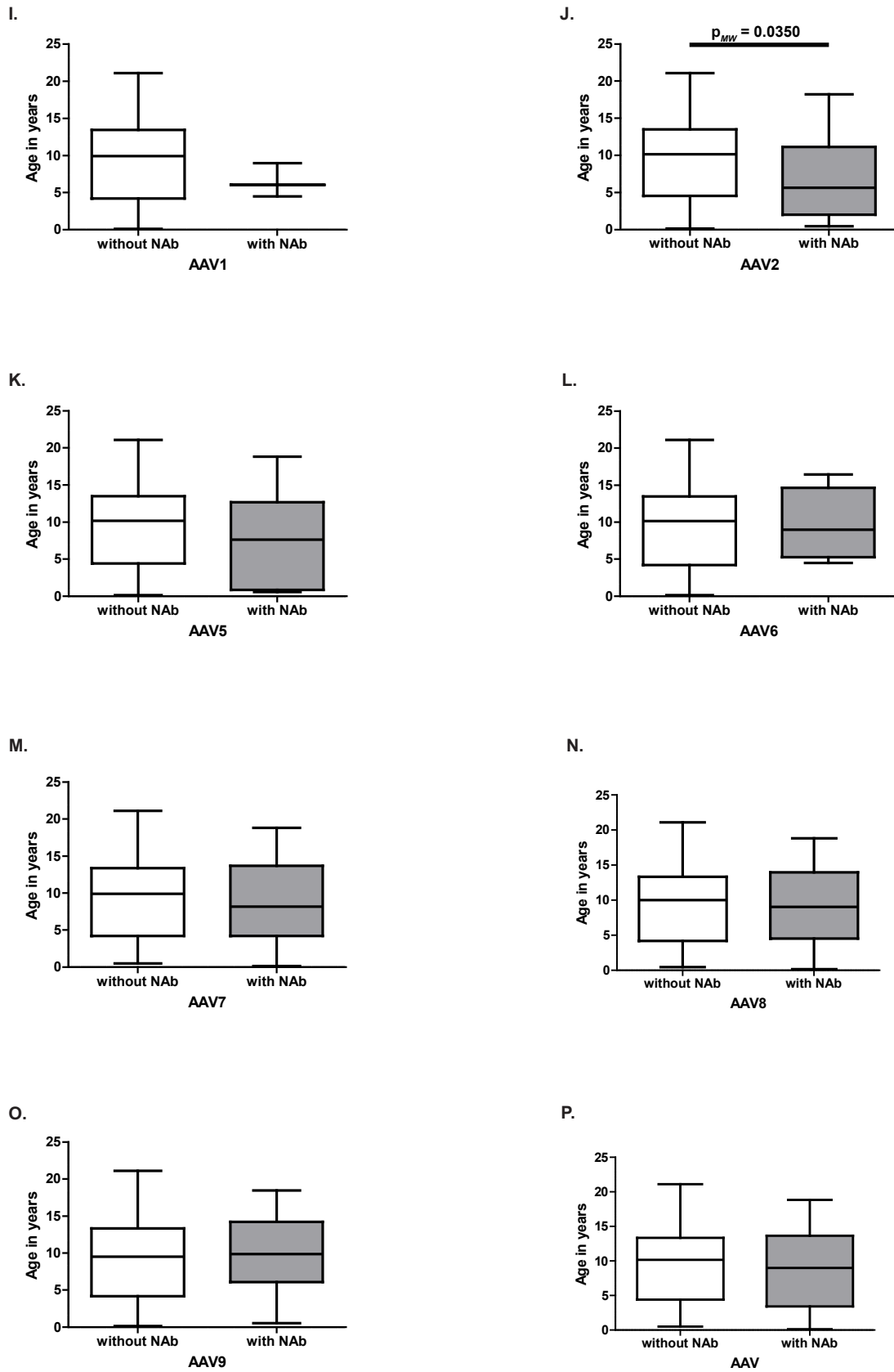

Q.

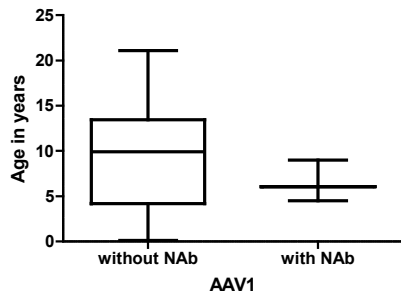

R.

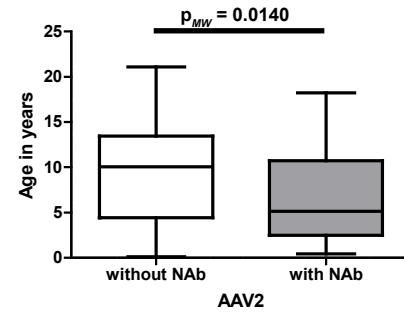

S.

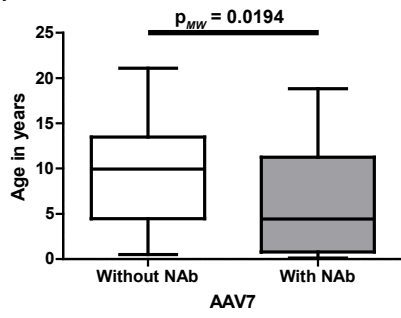

T.

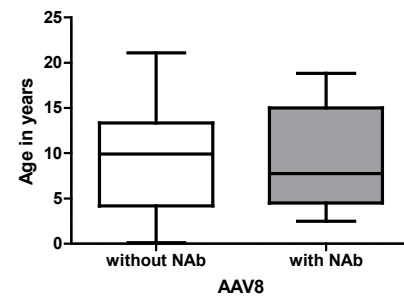

U.

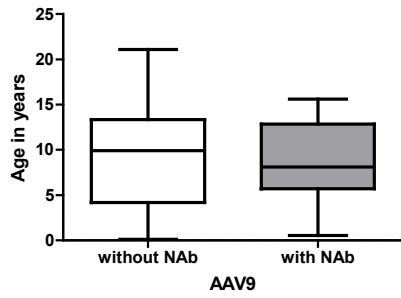

V.

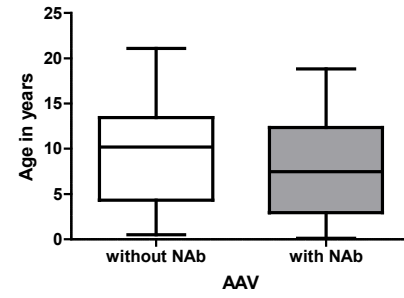

Serum Dilution:  $\geq 1:80$

W.

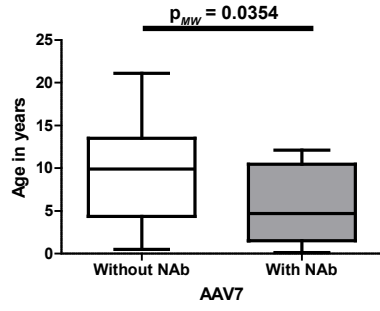

X.

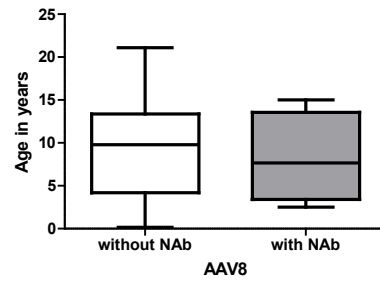

Y.

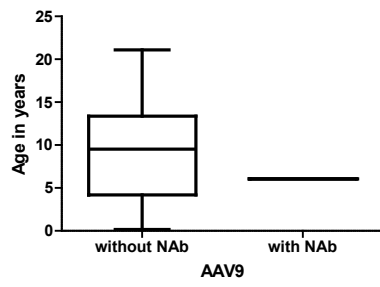

Z.

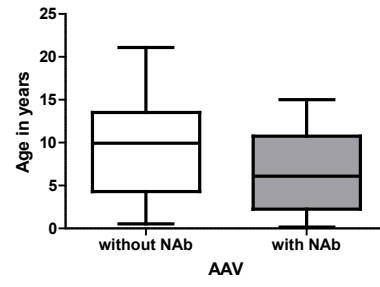

Serum Dilution:  $\geq 1:160$

AA.

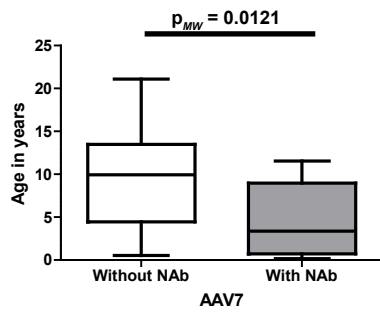

Supplement: S1 Fig — Shown here are comparisons of the ages of cats with and without NAb against AAV1, AAV2, AAV5, AAV6, AAV7, AAV8, AAV9 and all AAV serotypes combined, for the titers ≥1:10 (A-H), ≥1:20 (I-P), ≥1:40 (Q-V), ≥1:80 (W-Z) and ≥1:160 (AA). Samples were considered positive if the respective serum dilution inhibited transduction by ≥50%. A comparison of the ages of cats with and without NAb against each AAV serotype or all combined were analyzed using the Mann–Whitney U test (pMW). A p-value less than 0.05 was considered significant. The data are shown as box plots; the boxes extend from the 25th to the 75th percentile. The horizontal line represents the median, and the whiskers extend from the smallest to the largest value. (PDF) [file pone.0212811.s004.pdf]

Serum Dilution:  $\geq 1:10$

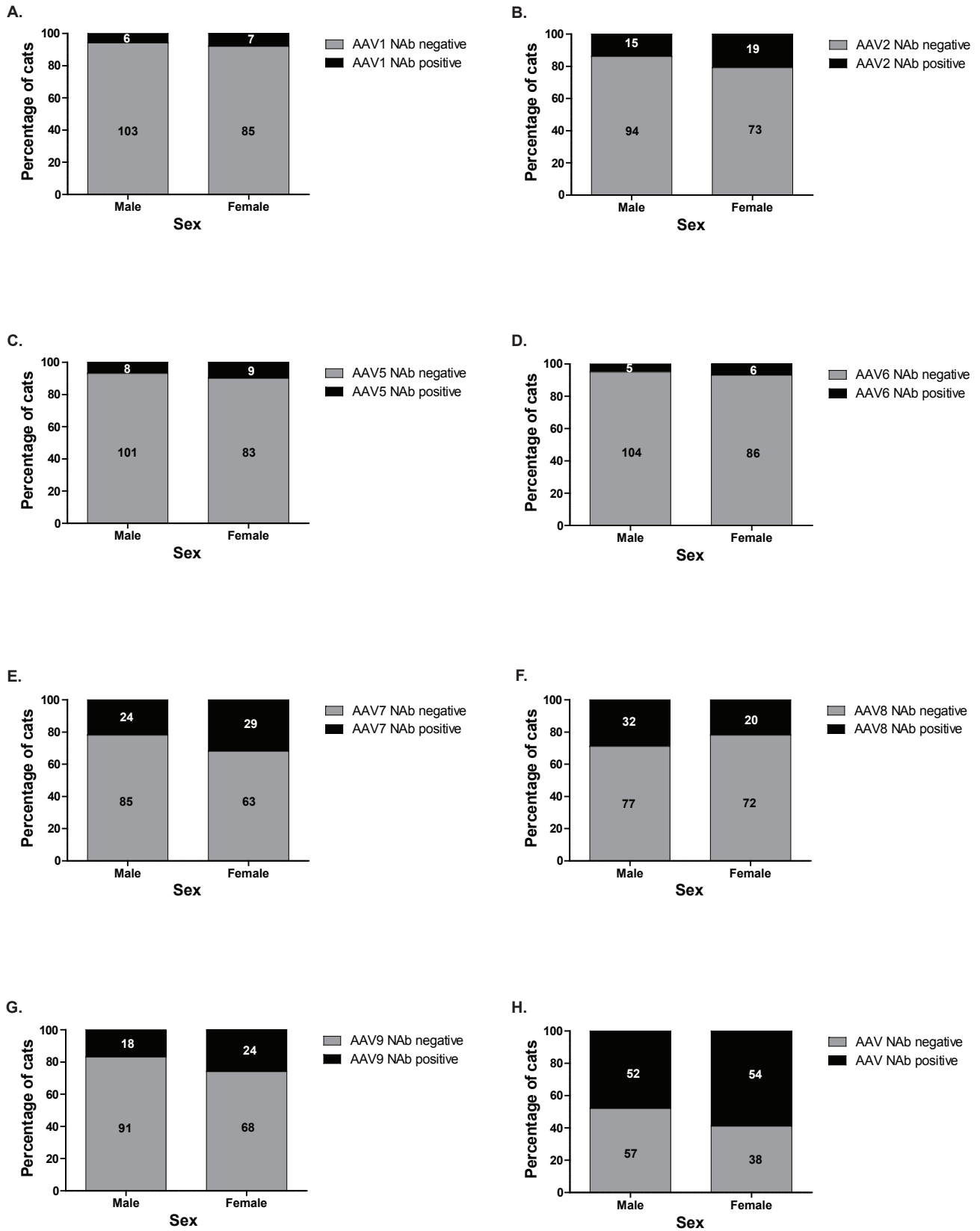

Serum Dilution:  $\geq 1:20$

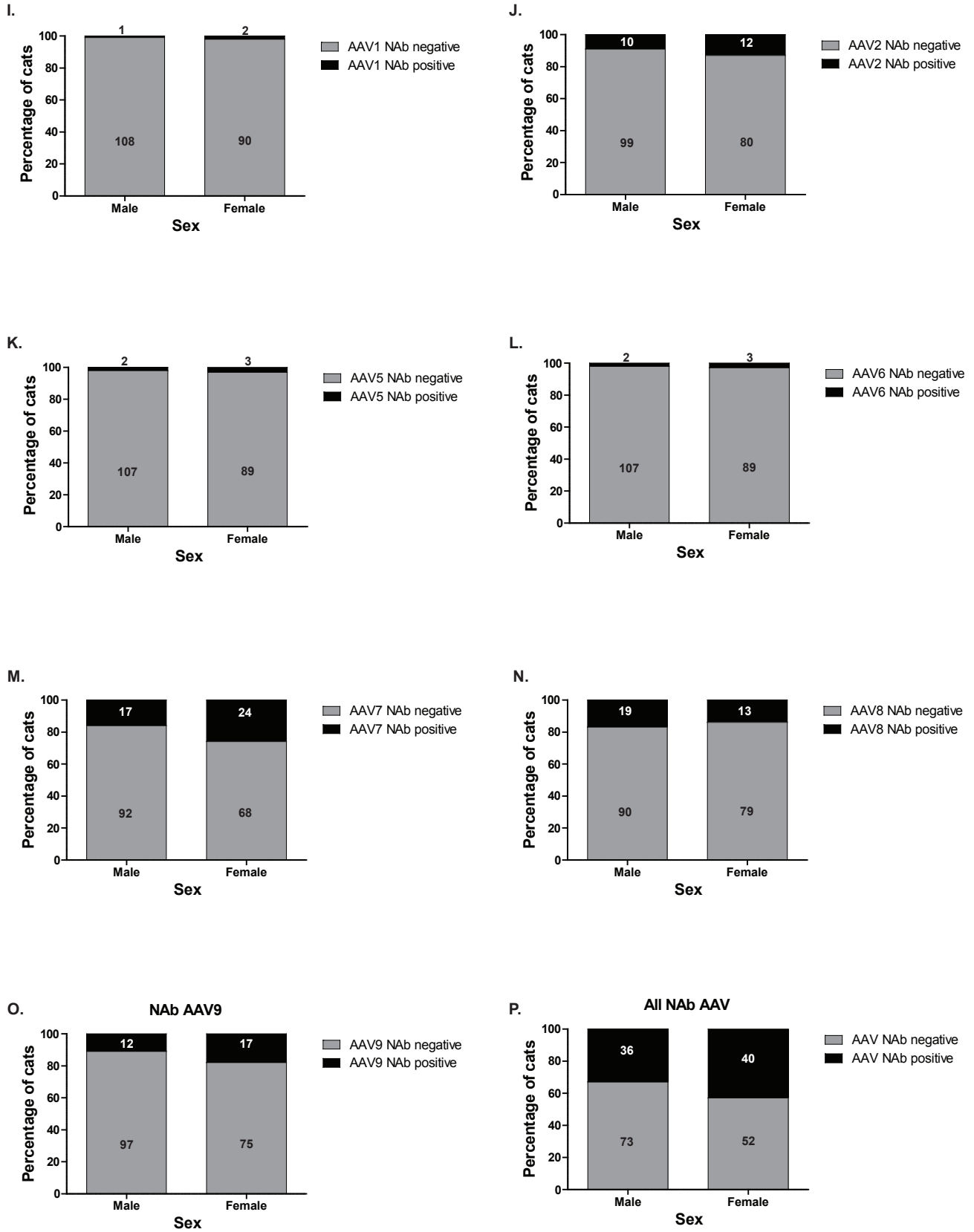

Serum Dilution:  $\geq 1:40$

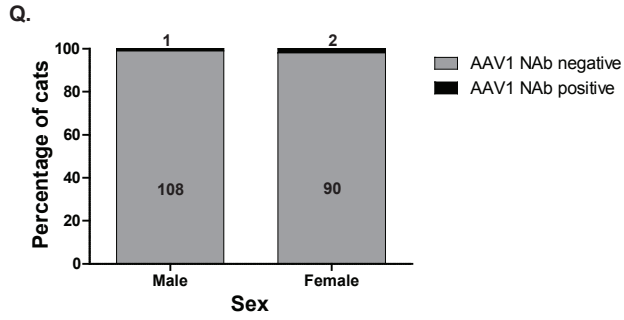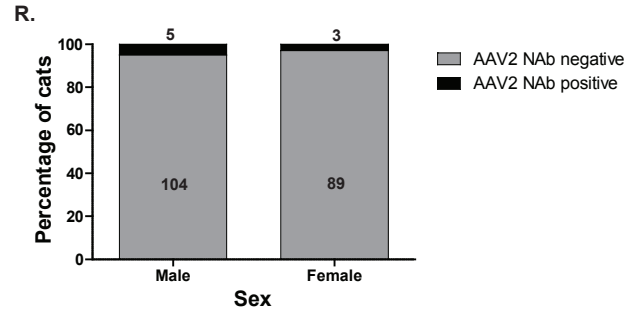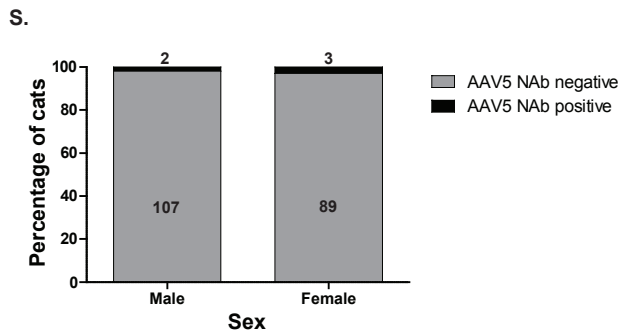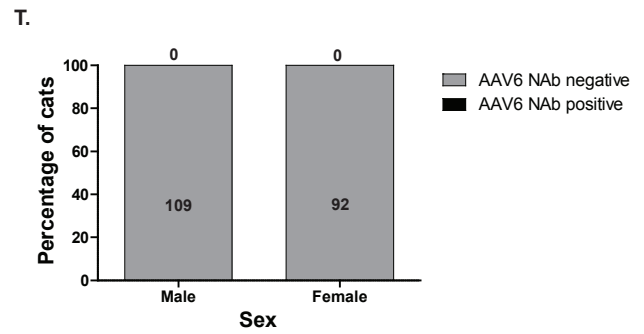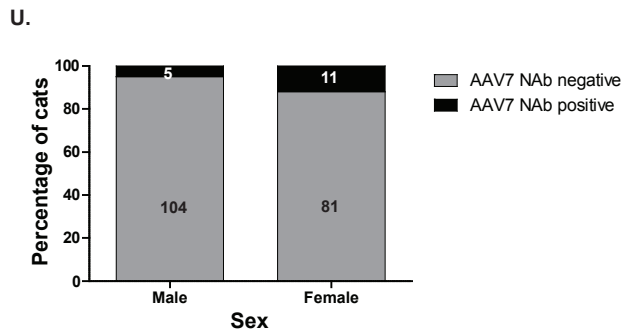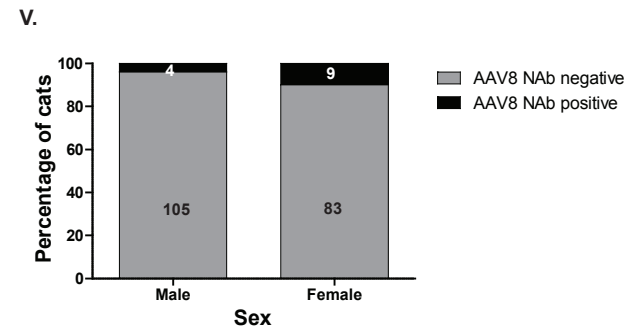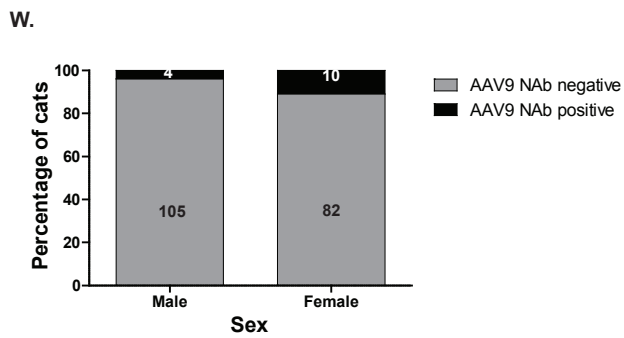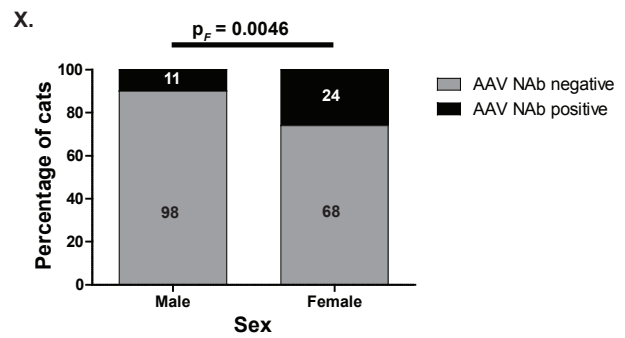

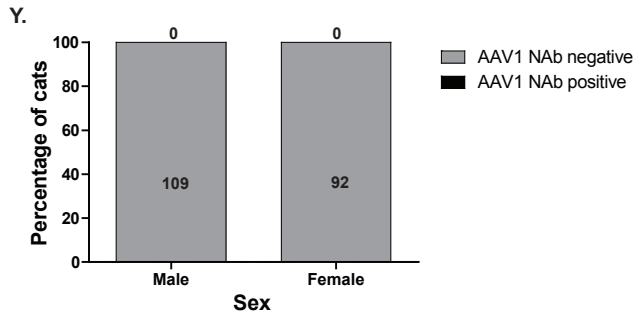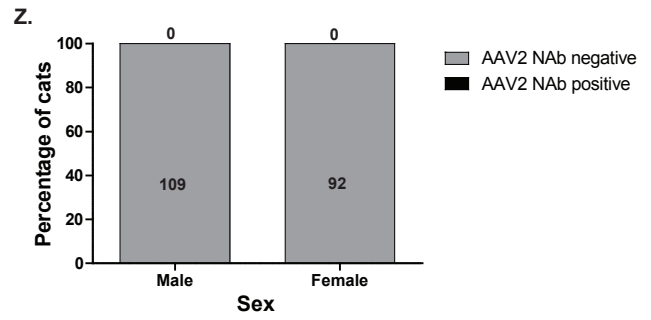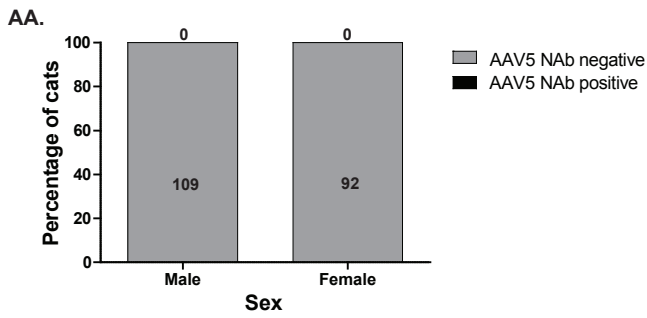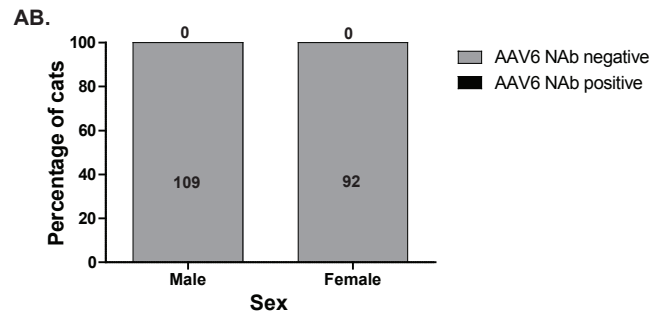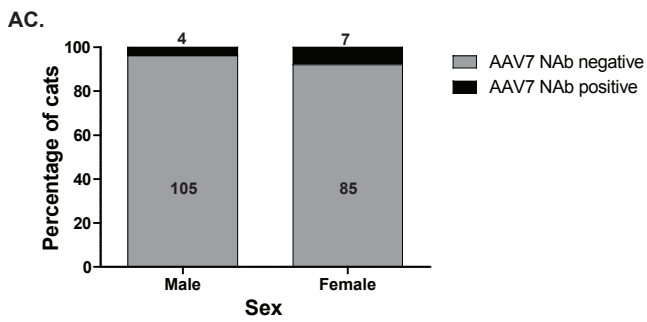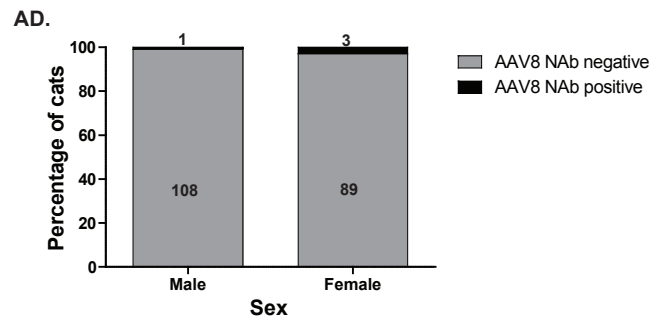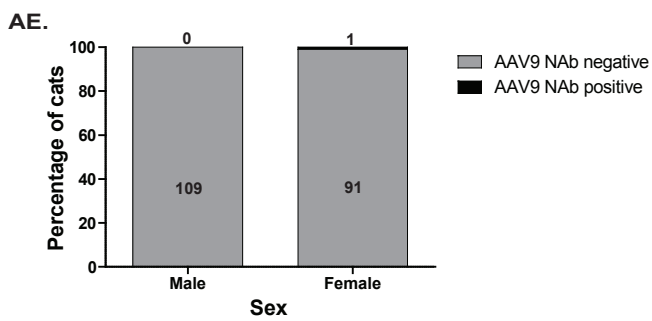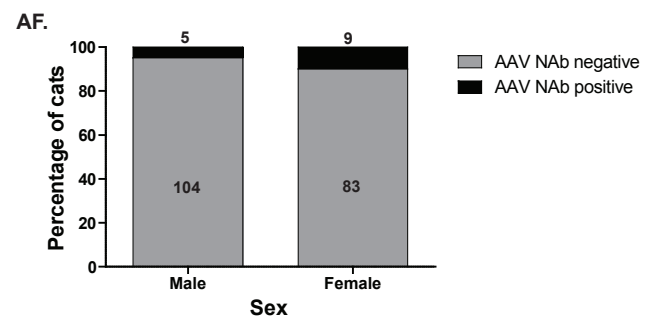

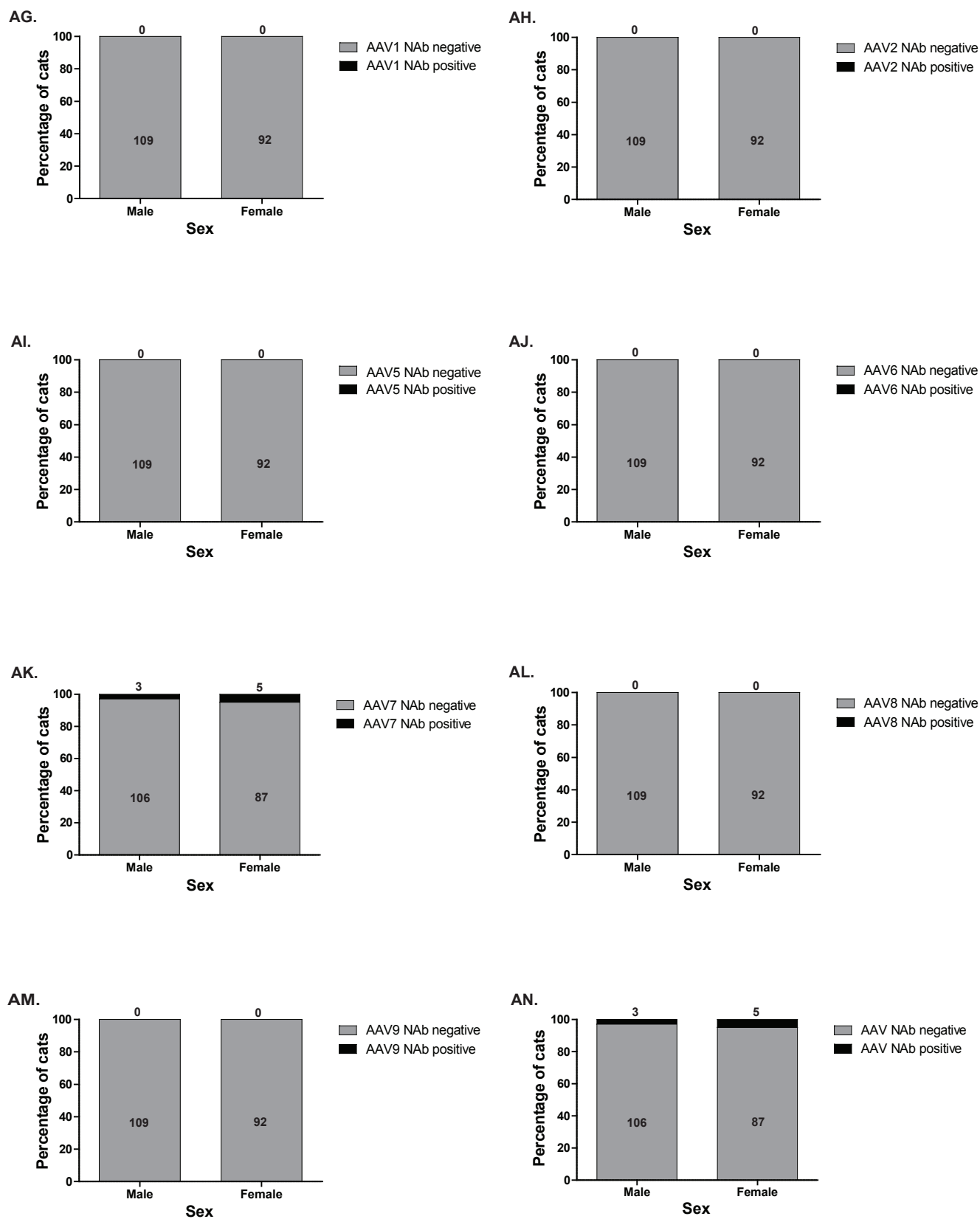

Supplement: S3 Fig — Cats are grouped by sex (male and female cats) and the presence or absence of NAb against AAV1, AAV2, AAV5, AAV6, AAV7, AAV8, AAV9 and all AAV serotypes combined, for the titers ≥1:10 (A-H), ≥1:20 (I-P), ≥1:40 (Q-X), ≥1:80 (Y-AF) and ≥1:160 (AG-AN). Frequencies were compared using Fisher’s exact test for small numbers (pF). A p-value less than 0.05 was considered significant. A statistically significant difference could be detected between sexes when the prevalence of NAb against all AAV serotypes combined was compared at a titer of ≥1:40 (pF > 0.0046, n = 201; 109 male and 92 female). Samples were considered positive if the respective serum dilutions inhibited in vitro transduction by ≥50%. (PDF) [file pone.0212811.s006.pdf]

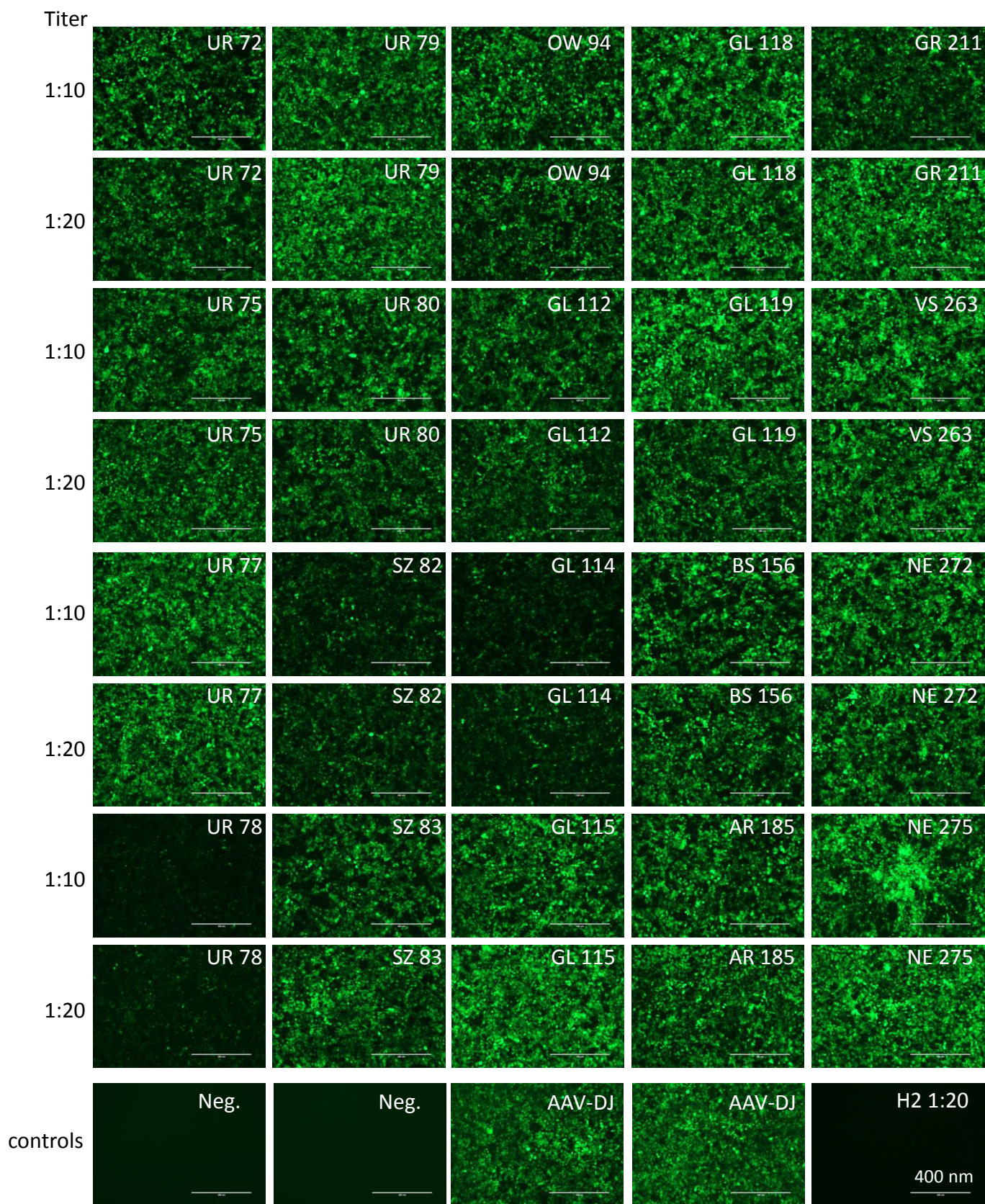

Supplement: S4 Fig — A transduction inhibition assay was performed in order to determine the presence of NAb against AAV-DJ-EGFP. Twenty cat serum samples previously shown to have NAb against other AAV serotypes were chosen. Two different serum dilutions were tested for each sample: 1:10 and 1:20. Controls included an AAV-NAb-positive human serum (H2 1:20), a non-serum control (AAV-DJ) and cells only (mock-infection without a virus; Neg.). Descriptions within each picture refer to the specific serum/plasma samples. The bar represents 400 nm. (PDF) [file pone.0212811.s007.pdf]
