# Supplementary material for: Pre-existing antibodies to candidate gene therapy vectors (adeno-associated vector serotypes) in domestic cats
Source: PLoS One. 2019 Mar 21;14(3):e0212811. doi: 10.1371/journal.pone.0212811 (PMC6428272; doi:10.1371/journal.pone.0212811)
Supplement: S2 Fig — Depicted here are the cats grouped as purebred (including Birmans, Ocicats, Siamese and others) or non-purebred (European Shorthairs and mixed breeds) and as having or lacking NAb against AAV1, AAV2, AAV5, AAV6, AAV7, AAV8, AAV9 and all AAV serotypes combined, for the titers ≥1:10 (A-H), ≥1:20 (I-P), ≥1:40 (Q-X), ≥1:80 (Y-AF) and ≥1:160 (AG-AN). Frequencies were compared using Fisher’s exact test for small numbers (pF). A p-value less than 0.05 was considered significant. No statistically significant difference could be detected when the prevalence of NAb against various AAV serotypes was compared between European Shorthairs (and mixed breeds) and other cat breeds (pF > 0.05) at a titer of ≥1:10. The numbers within the columns represent the numbers of cats included in the analysis. A total of 56 purebred and 125 non-purebred cats were included. Samples were considered positive if the respective serum dilutions inhibited transduction by ≥50%. (PDF) [file pone.0212811.s005.pdf]

Serum Dilution: ≥ 1:10

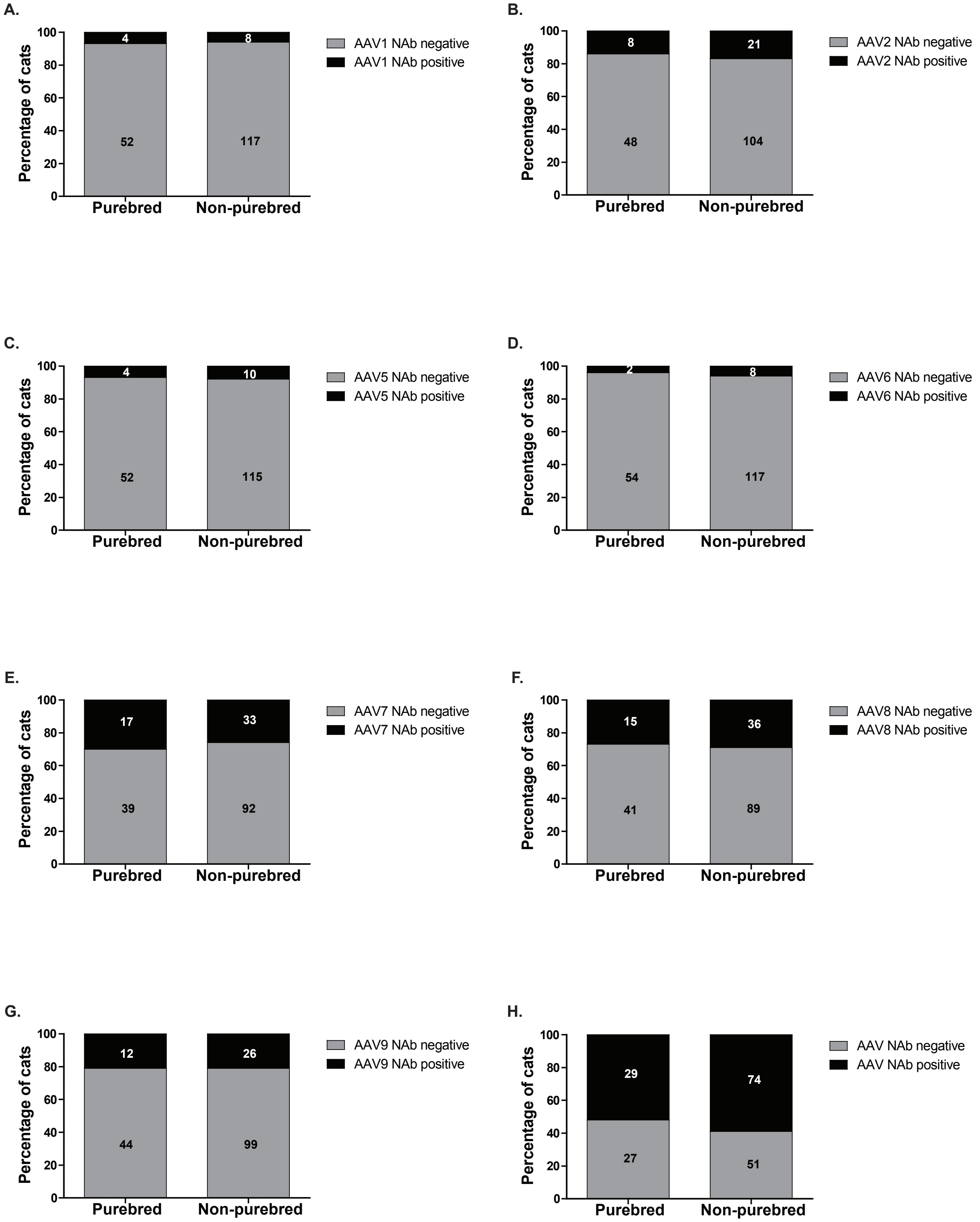

Li et al., S2 Fig

Serum Dilution: ≥ 1:20

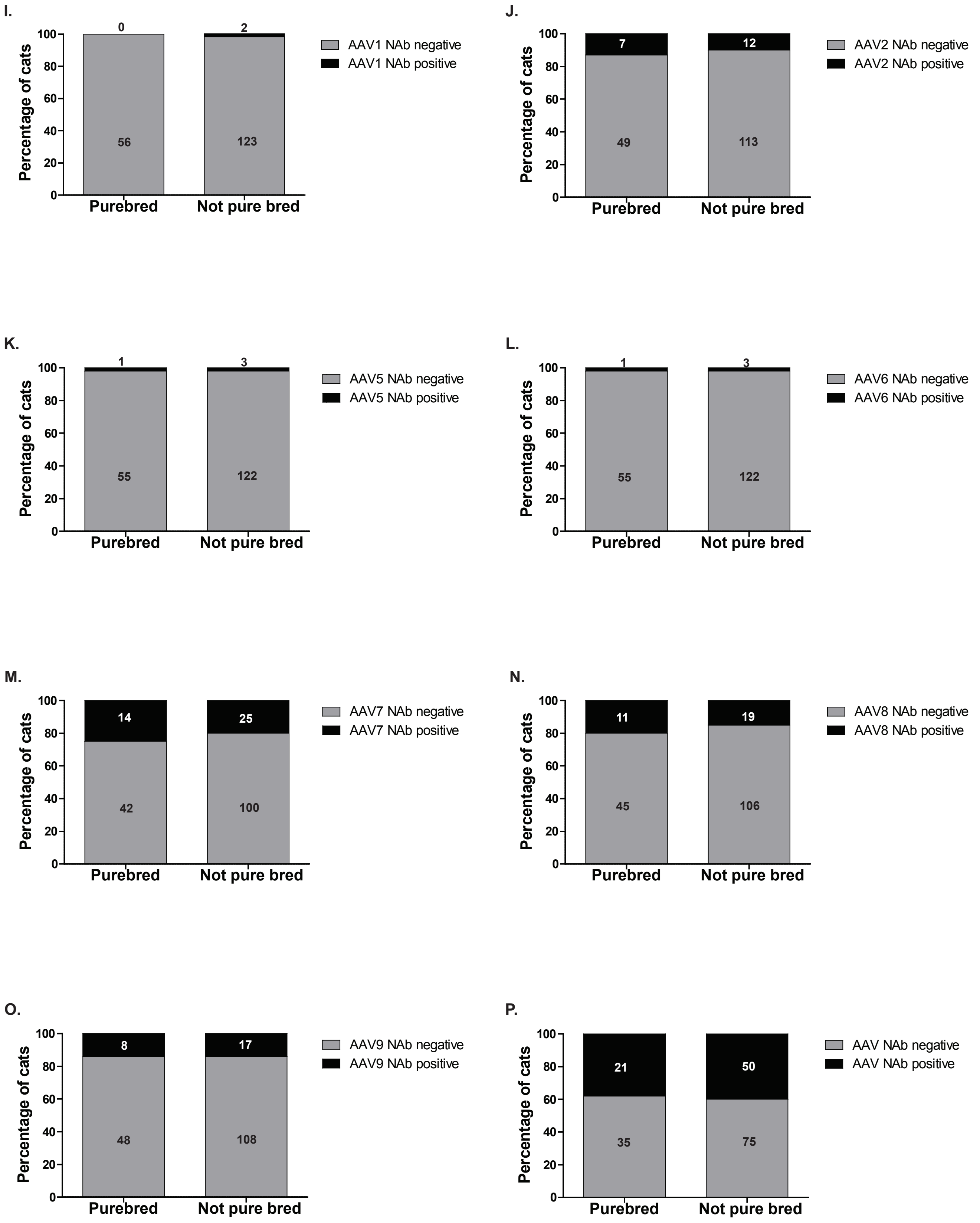

Li et al., S2 Fig b

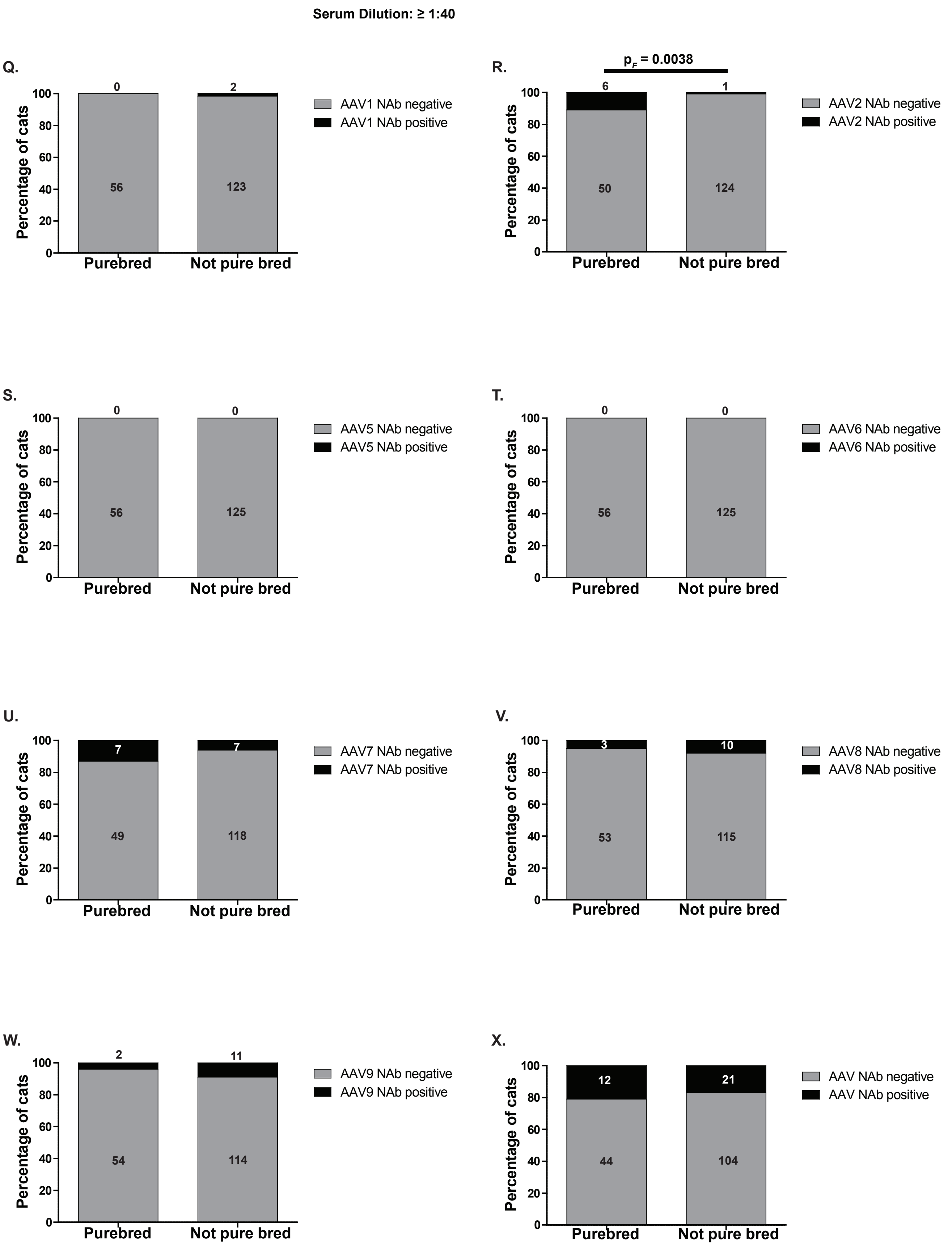

Li et al., S2 Fig c

Serum Dilution: ≥ 1:80

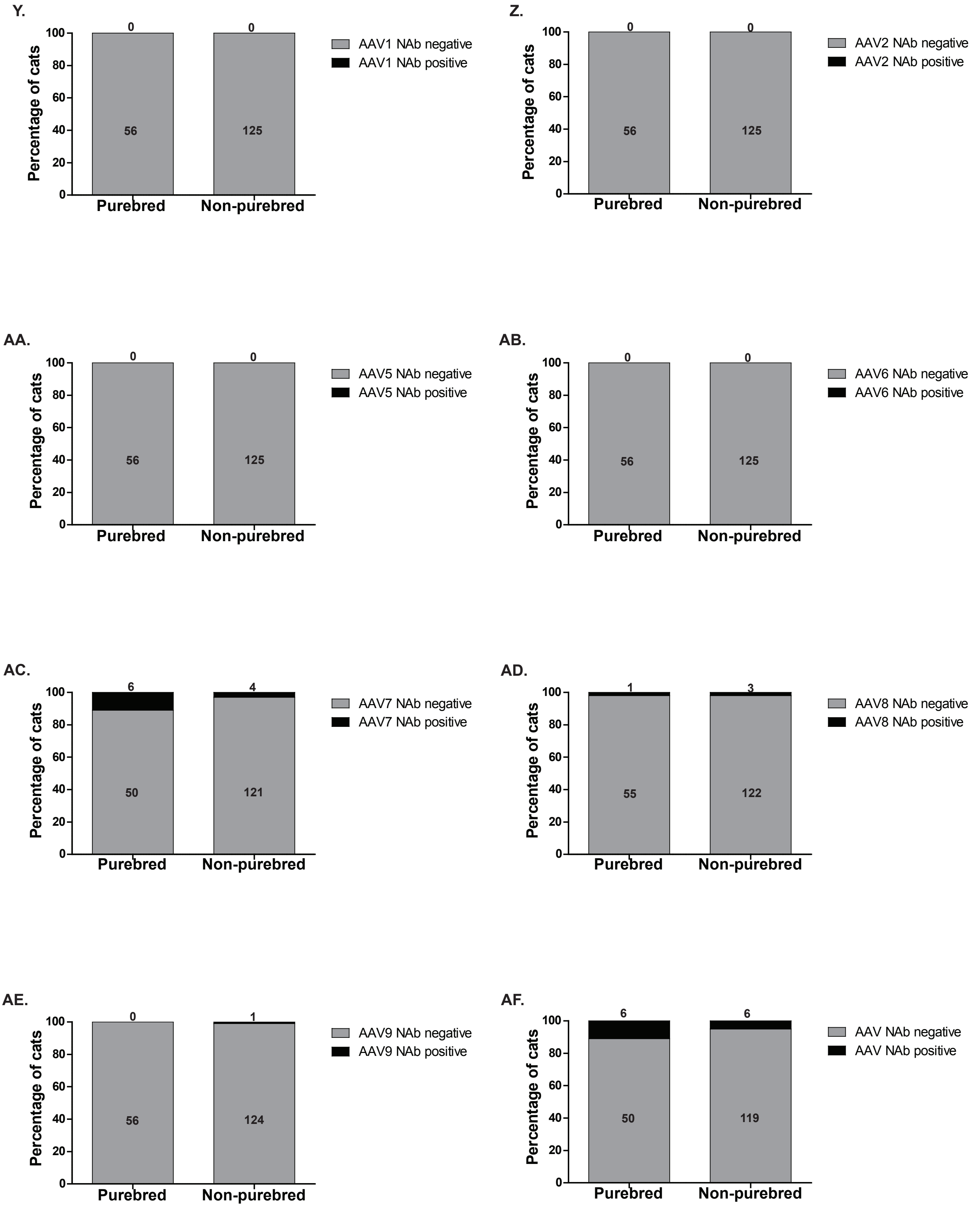

Li et al., S2 Fig d

Serum Dilution: ≥ 1:160

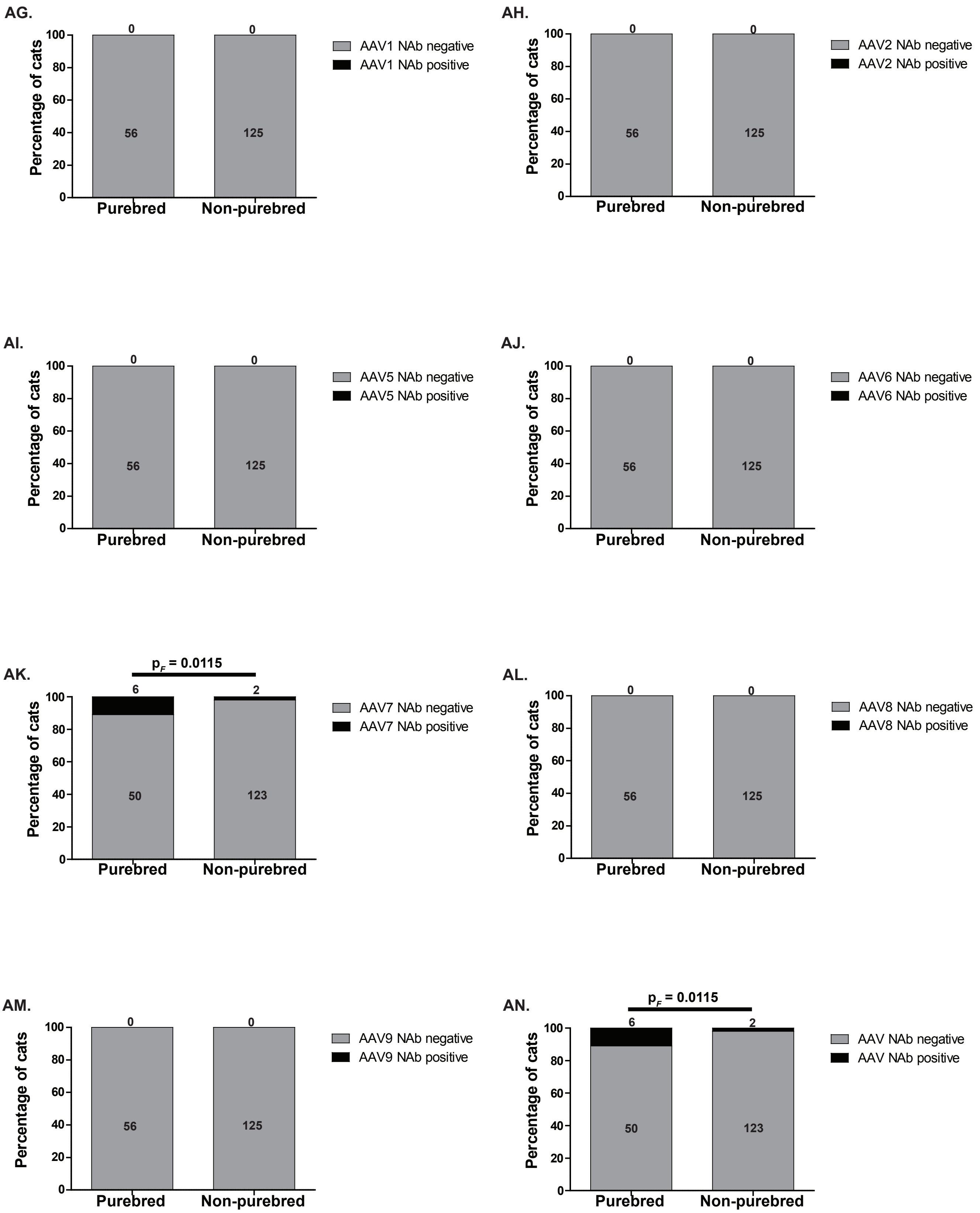

Li et al., S2 Fig e
