# Supplementary material for: Pre-existing antibodies to candidate gene therapy vectors (adeno-associated vector serotypes) in domestic cats
Source: PLoS One. 2019 Mar 21;14(3):e0212811. doi: 10.1371/journal.pone.0212811 (PMC6428272; doi:10.1371/journal.pone.0212811)
Supplement: S1 File — (DOCX) [file pone.0212811.s008.docx]

# S1 File

## AAV-DJ-EGFP vector

## In the present study, an ssAAV-DJ/2-hCMV-chI-EGFP-WPRE-SV40p(A) vector was used to test for NAb against AAV-DJ. This vector contained enhanced green fluorescent protein (EGFP) as a reporter, driven by a cytomegalovirus (CMV) promoter. This vector was constructed, purified and quantified by the Viral Vector Facility (VVF, University of Zurich, Zurich, Switzerland).

## NAb assay

In the present study, NAb against AAV-DJ were investigated by using an *in vitro* transduction inhibition assay. HEK-293T cells, seeded at 5 × 10^4^ cells per well, were incubated for 24 hours. Cat serum or plasma samples were heat inactivated at 56 °C for 35 minutes before being diluted with Advanced RPMI 1640 to a final concentration of 1:10 or 1:20. Diluted serum/plasma (100 μl) was mixed with AAV-DJ at an MOI of 500 and incubated at 37 °C for 1 hour before being added to the cells. One hour later, 100 µl of Advanced RPMI 1640 containing 20% FCS was added to each well and incubated for three days at 37 °C. As controls, wells with AAV-NAb-positive human serum, a non-serum control and a mock-infection (blank) were included in the transduction inhibition assay.

After cultivation, the cells were microscopically investigated for the presence of a signal. Inhibition was confirmed if AAV-DJ-EGFP transduction was profoundly reduced compared to the non-serum control.

## Immunofluorescence assay (IFA)

The serum sample of the AAV NAb-positive cat (QLK1) was tested for the presence of antibodies to feline herpes virus 1 (FHV-1), feline calicivirus (FCV), and feline parvovirus (FPV) with the indirect immunofluorescence assay ([1](#_ENREF_1), [2](#_ENREF_2)). The serum sample was diluted 1:20, 1:40, 1:80, 1:160 and 1:320 in 1xPBS and added on slides coated with CrFK cells infected with a viral field strain (FHV-1), or viral vaccine strains (FCV, FPV). As controls, defined positive and negative feline reference serum samples were used. After incubation of 1 hour and several washing steps, a rabbit anti-cat IgG/FITC (Nordic MUbio, Susteren, Netherlands) diluted 1:40 in 1xPBS was added. After 1 hour of incubation, the fluorescence signal was detected using a fluorescence microscope (Leica DMLB, Heerbrugg, Switzerland). Results were expressed as titers (S3 Table).

**References**

1. Hofmann-Lehmann R, Fehr D, Grob M, Elgizoli M, Packer C, Martenson JS, et al. Prevalence of antibodies to feline parvovirus, calicivirus, herpesvirus, coronavirus and immunodeficiency virus and of feline leukemia virus antigen and the interrelationship of these viral infections in free-ranging lions in East Africa. Clinical and Diagnostic Laboratory Immunology. 1996;3(5):554-62.

2. Filoni C, Catao-Dias JL, Bay G, Durigon EL, Jorge RS, Lutz H, et al. First evidence of feline herpesvirus, calicivirus, parvovirus, and Ehrlichia exposure in Brazilian free-ranging felids. J Wildl Dis. 2006 Apr;42(2):470-7.
